# Arene Variation of Highly Cytotoxic Tridentate Naphthoquinone-Based Ruthenium(II) Complexes and In-Depth In Vitro Studies

**DOI:** 10.3390/pharmaceutics14112466

**Published:** 2022-11-15

**Authors:** Klaudia Cseh, Heiko Geisler, Kristina Stanojkovska, Julia Westermayr, Philipp Brunmayr, Dominik Wenisch, Natalie Gajic, Michaela Hejl, Martin Schaier, Gunda Koellensperger, Michael A. Jakupec, Philipp Marquetand, Wolfgang Kandioller

**Affiliations:** 1 Institute of Inorganic Chemistry, Faculty of Chemistry, University of Vienna, Waehringer Str. 42, 1090 Vienna, Austria; 2 Institute of Theoretical Chemistry, Faculty of Chemistry, University of Vienna, Waehringer Str. 17, 1090 Vienna, Austria; 3 Wilhelm-Ostwald-Institute for Physical and Theoretical Chemistry, Faculty of Chemistry and Mineralogy, University of Leipzig, Linnéstr. 2, 04103 Leipzig, Germany; 4 Institute of Analytical Chemistry, Faculty of Chemistry, University of Vienna, Waehringer Str. 38, 1090 Vienna, Austria; 5 Vienna Doctoral School in Chemistry (DoSChem), University of Vienna, Waehringer Str. 42, 1090 Vienna, Austria; 6 Research Cluster “Translational Cancer Therapy Research”, University of Vienna, 1090 Vienna, Austria; 7 Vienna Research Platform on Accelerating Photoreaction Discovery, University of Vienna, 1090 Vienna, Austria

**Keywords:** metal-based drugs, ruthenium, piano-stool complexes, anticancer

## Abstract

The main purpose of this study was to synthesize a new set of naphthoquinone-based ruthenium(II) arene complexes and to develop an understanding of their mode of action. This study systematically reviews the steps of synthesis, aiming to provide a simplified approach using microwave irradiation. The chemical structures and the physicochemical properties of this novel group of compounds were examined by ^1^H-NMR and ^13^C-NMR spectroscopy, X-ray diffractometry, HPLC-MS and supporting DFT calculations. Several aspects of the biological activity were investigated in vitro, including short- and long-term cytotoxicity tests, cellular accumulation studies, detection of reactive oxygen species generation, apoptosis induction and NAD(P)H:quinone oxidoreductase 1 (NQO1) activity as well as cell cycle analysis in A549, CH1/PA-1, and SW480 cancer cells. Furthermore, the DNA interaction ability was studied in a cell-free assay. A positive correlation was found between cytotoxicity, lipophilicity and cellular accumulation of the tested complexes, and the results offer some important insights into the effects of the arene. The most obvious finding to emerge from this study is that the usually very chemosensitive CH1/PA-1 teratocarcinoma cells showed resistance to these phthiocol-based organometallics in comparison to the usually less chemosensitive SW480 colon carcinoma cells, which pilot experiments suggest as being related to NQO1 activity.

## 1. Introduction

Platinum-based compounds are an important pillar in the fight against cancer, particularly the worldwide-approved drugs cisplatin, carboplatin and oxaliplatin [1,2]. However, these drugs cause severe side effects such as nephrotoxicity, myelosuppression and neurotoxicity [3]. To overcome these drawbacks, scientists worldwide are searching for alternatives, such as small-molecule receptor tyrosine kinase inhibitors (e.g., sunitinib), monoclonal antibodies (e.g., cetuximab, bevacizumab, ipilimumab), CAR-T cells, vaccines, oncolytic viruses and new cytostatic drugs (e.g., novel platinum-based compounds) [4,5,6]. Since the latter interfere with cell proliferation, not only cancer cells but also normal cells are affected, which leads to severe side effects.

**Figure 1:**
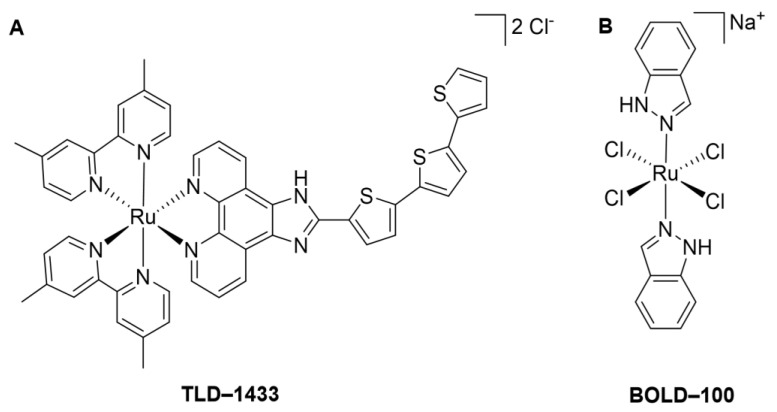
Chemical structures of clinically studied ruthenium compounds. Ru(II) coordination compound TLD–1433 (**A**) and Ru(III) drug BOLD–100 (**B**).

One line of research is the use of ruthenium instead of platinum, due to the lower toxicity of active complexes [7]. There are two prominent ruthenium coordination compounds which have shown promising results in (pre)clinical trials, namely TLD–1433 and BOLD–100 (formerly KP1139, IT–139). The ruthenium(II) compound TLD–1433 (Figure 1A) is under investigation for photodynamic therapy and a clinical phase two trial in patients with non-muscle invasive bladder cancer is in progress (ClinicalTrials.gov, Identifier: NCT03945162) [8]. The ruthenium(III) drug BOLD-100 (Figure 1B) was granted an Orphan Drug Designation (ODD) in pancreatic cancer and a clinical trial of a combination therapy with FOLFOX (5-fluorouracil + folinic acid + oxaliplatin) in gastro-intestinal cancers is ongoing (ClinicalTrials.gov Identifier: NCT04421820) [9].

However, not only coordination compounds of ruthenium are in the focus of metal-based anticancer drug research, but also organometallic ruthenium(II) compounds. The best-studied compounds are RAPTA-C from Dysons’ group, which mainly interacts with proteins, and RM175 from Sadlers’ group, which mainly interferes with DNA [10]. This complex class exhibits a piano-stool structure, in which the metal center is surrounded by an arene and variable ligands with different denticities. The three coordination sites can be occupied by three monodentate, one bidentate and one monodentate, or one tridentate ligand. This pseudo-octahedral geometry provides a powerful tool for controlling pharmacokinetic and pharmacodynamic properties. Each building block of the complex can influence chemical and biological properties, such as stability, cytotoxicity, cellular uptake, electrochemical behavior, and hydrolysis [11,12,13]. Furthermore, bioactive ligands were often used in order to increase the cytotoxic effect or for targeting.

One interesting compound class is naphthoquinones, which demonstrate several potential modes of action, such as ROS (reactive oxygen species) generation, apoptosis induction through ER (endoplasmic reticulum) stress and inhibition of topoisomerase, as well as interference with p53 or NAD(P)H:quinone oxidoreductase 1 activity (NQO1) [14].

**Figure 2:**
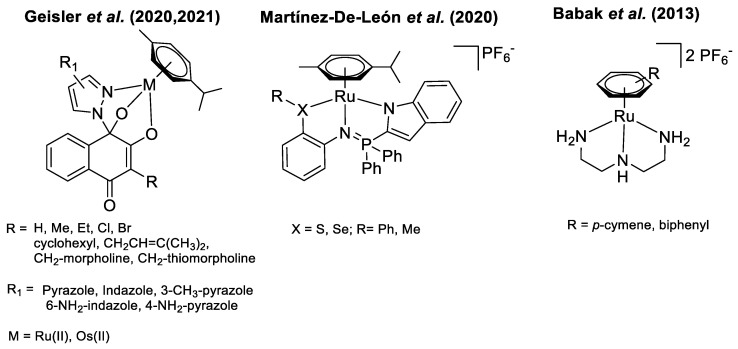
Tridentate ruthenium arene complexes with anticancer properties [17,18,19,20].

Due to this broad range of properties favoring anticancer activity, naphthoquinone-based organometallics were developed and investigated. Complexes with a labile chlorido-leaving group have shown promising results in vitro and in vivo [15,16]. However, low stability in an aqueous solution may explain the occurrence of severe side effects during in vivo experiments. Stability was improved by an in situ formation of a hemiaminal bond between an azole and a naphthoquinone moiety, yielding complexes with a tridentate ligand, which increased the stability enormously [17]. Only a few examples of tridentate ligands are reported in the literature for anticancer drug research (Figure 2). The *N*, *N*, *N*-coordinated ruthenium complex from Babak et al. has shown high stability, which leads to inertness against biomolecules resulting in low activity in vitro [18]. However, our group synthesized novel tridentate naphthoquinone complexes, providing highly cytotoxic and more stable *N*, *O*, *O*-complexes compared to the parental bidentate chlorido compounds [17,19]. Furthermore, Martinez-De-León et al. published cationic *N*, *N*, *S*- and *N*, *N*, *Se*-iminophosphorane organometallics with good cytotoxicity in several cancer cell lines [20].

Within this work, we synthesized, characterized and investigated phthiocol-based organometallics with different arenes and their stabilities at physiological pH by HPLC-MS measurements. Density Functional Theory (DFT) calculations were performed to prove the stability and hydrolysis hypotheses. In addition, the most relevant aspects of their biological activity were investigated in several biological assays, such as determination of cytotoxicity, cellular accumulation, ROS generation ability, apoptosis induction, DNA interaction, as well as influence on the cell cycle and NQO1 activity.

## 2. Materials and Methods

The used dimeric metal precursors [Ru(*p*-cymene)Cl_2_]_2_, [RuCl_2_(benzene)]_2_, [RuCl_2_(toluene)]_2_, [RuCl_2_(indane)]_2_ and [RuCl_2_(biphenyl)]_2_ were synthesized according to the literature [21,22,23]. Phthiocol (**L**) [24,25] and complex **1** [17]) were synthesized as reported. The following chemicals were used without further purification for the syntheses: RuCl_3_ × H_2_O (Johnson Matthey, London, UK), biphenyl, cyclohexa-1,3-diene, indane, sodium (Sigma-Aldrich, St. Louis, MO, USA), 1-methyl-1,4-cyclohexadiene, α-terpinene (Alfa Aesar, Haverhill, MA, USA), 1*H*-pyrazole, triethylamine, menadione (Acros-Fisher, Waltham, MA, USA), [RuCl_2_(hexamethylbenzene)]_2_ (TCI, Tokyo, Japan) and hydrogen peroxide (30%, VWR, Radnor, PA, USA).

Solvents were purchased from commercial suppliers and dried before use if needed. Microwave reactions were performed with a Biotage^®^ Initiator+ system (Uppsala, Sweden). Purification via flash column chromatography was conducted with Biotage^®^ Isolera system (Uppsala, Sweden) and silica gel (VWR, mesh 40–63 µm). Elemental analyses were performed by the Microanalytical Laboratory of the University of Vienna with a Eurovector EA 3000(2009), equipped with a high-temperature pyrolysis furnace (HT, Hekatech, Osnabrück Germany, 2009). Elemental analysis samples were weighed on a Sartorius SEC 2 ultra-micro balance with ±0.1 µg resolution. Sample weights from 1–3 mg were used. For calibration, two NIST-certified reference materials were used: sulfanilamide (C_6_H_8_N_2_O_2_S) and BBOT (2, 5-bis-(5-tert-butyl-2-benzoxazole-2-yl)-thiophenone, C_26_H_26_N_2_O_2_S). The limit of quantification (LOQ) was 0.05 w-% for C, H, N and 0.02 w-% for S. For samples without N and/or S, the content of these elements was determined and verified to be below LOQ. The presented values are the average of determinations in triplicate. The general numbering of carbons and hydrogens and the corresponding NMR spectra of complexes **2**–**6** were described in the SI (Figures S1–S10). ^1^H-,^13^C-and 2D-NMR spectra were recorded at 298 K on a Bruker AV III 600 or AV NEO 500 spectrometers at 600.25/500.10 MHz (^1^H) and 150.95 MHz (^13^C) (Bruker, Billerica, MA, USA). Single crystals of four compounds (**2**–**4**, **6**) were obtained by vapor diffusion or liquid–liquid diffusion method from dichloromethane/diethyl ether or dichloromethane/*n*-hexane. The X-ray intensity data were measured on a Bruker D8 Venture diffractometer equipped with a multilayer monochromator, Mo and Cu K/α INCOATEC microfocus sealed tube and Oxford cooling system (Bruker, Billerica, MA, USA).

### 2.1. Synthesis

#### General Procedure for Complex Syntheses (**2**–**6**) [17]

The corresponding dimer (0.10 mmol), 1*H*-pyrazole (0.19–0.21 mmol), NEt_3_ (0.6 mmol) and the desired phthiocol (**L**) (0.19–0.21 mmol) were dissolved in 8–12 mL methanol and stirred at 50–60 °C for 20–30 minutes under microwave irradiation. The solvent was removed, and the residue was purified by flash chromatography (SiO_2_) with a ternary eluent system (EtOAc/*n*-hexane/NEt_3_ or EtOAc/MeCN/NEt_3_). The fractions were combined, evaporated and dried in vacuo. Oily residues were dissolved in dichloromethane, precipitated with n-hexane, separated and washed with *n*-hexane, and dried in vacuo.

[3-Methyl-4-oxo-(1H-κ*N*^2^-pyrazol-1-yl)-1,4-dihydronaphtalene-1,2-bis(olato)-κ*O*^1^-κ*O*^2^)(*η*^6^-benzene)ruthenium(II)](**2**)

The reaction was performed according to the general procedure using [RuCl_2_(benzene)]_2_ (150 mg, 0.30 mmol), 1*H*-pyrazole (39 mg, 0.57 mmol), phthiocol (107 mg, 0.57 mmol) and NEt_3_ (251 µL, 182 mg, 1.80 mmol). The mixture was stirred at 60 °C for 20 minutes under microwave irradiation. Flash chromatography with EtOAc/MeCN/NEt_3_ (88/10/2). Yield: 93 mg yellow/green solid (0.21 mmol, 38%). Elemental analysis found: C, 52.61; H, 4.03; N, 6.12; O, 14.23. Calc. for C_20_H_16_N_2_O_3_Ru·(H_2_O)_1.1_ C, 53.00; H, 4.05; N, 6.18; O, 14.47%. ^1^H NMR (600.25 MHz, MeOD-d_4_) δ 8.40 (dd, *J* = 2.1, 0.6 Hz, 1H, H3′), 8.12–8.08 (m, 1H, H5–8), 7.65–7.59 (m, 3H, H5–8), 6.70 (d, *J* = 2.0 Hz, 1H, H5′), 6.35 (dd, *J* = 2.4, 2.4 Hz, 1H, H4′), 5.93 (s, 5H, Harene) and 1.74 (s, 3H, H9). ^13^C NMR (150.93 MHz, MeOD-d_4_) δ 184.2 (C4), 182.3 (C2), 142.0 (C3′), 137.4 (C4a/8a), 134.0 (C4a/8a), 132.5 (C5–8), 131.4 (C5–8), 128.0 (C5′), 127.6 (C5–8), 127.2 (C5–8), 108.8 (C4′), 105.8 (C3), 94.9 (C1), 82.9 (Carene) and 8.12 (C9).

[3-Methyl-4-oxo-(1*H*-κ*N*^2^-pyrazol-1-yl)-1,4-dihydronaphtalene-1,2-bis(olato)-κ*O*^1^-κ*O*^2^)(*η*^6^-toluene)ruthenium(II)](**3**)

The reaction was performed according to the general procedure using [RuCl_2_(toluene)]_2_ (150 mg, 0.28 mmol), 1*H*-pyrazole (36 mg, 0.53 mmol), phthiocol (100 mg, 0.53 mmol) and NEt_3_ (234 µL, 170 mg, 1.68 mmol). The mixture was stirred at 50 °C for 20 minutes under microwave irradiation. Flash chromatography with EtOAc/*n*-hexane/NEt_3_ (90/5/5). Yield: 117 mg yellow solid (0.26 mmol, 49%). Elemental analysis found: C, 54.62; H, 4.20; N, 6.27; O, 12.31. Calc. for C_21_H_18_N_2_O_3_Ru·(H_2_O)_0.7_: C, 54.82; H, 4.25; N, 6.09; O, 12.87%. ^1^H NMR (600.25 MHz, MeOD-d_4_) δ 8.34 (dd, *J* = 2.2, 0.6 Hz, 1H, H3′), 8.12–8.08 (m, 1H, H5–8), 7.65–7.58 (m, 3H, H5–8), 6.69 (dd, *J* = 2.6, 0.6 Hz, 1H, H5′), 6.34 (t, *J* = 2.4 Hz, 1H, H4′), 6.12 (dd, *J* = 5.7, 5.7 Hz, 1H, Hc), 5.98 (dd, *J* = 5.7, 5.7 Hz, 1H, Hc), 5.66 (d, *J* = 5.8 Hz, 1H, Hb), 5.64 (dd, *J* = 5.6, 5.6 Hz, 1H, Hd), 5.57 (d, *J* = 5.6 Hz, 1H, Hb), 2.37 (s, 3H, He) and 1.75 (s, 3H, H9). ^13^C NMR (150.95 MHz, MeOD-d_4_) δ: 184.0 (C4), 183.0 (C2), 141.8 (C3′), 137.6 (C4a/8a), 134.0 (C4a/8a), 132.4 (C5–8), 131.3 (C5–8), 128.0 (C5′), 127.7 (C5–8), 127.2 (C5–8), 108.7 (C4′), 105.7 (C3), 101.1 (Ca), 94.9 (C1), 86.2 (Cc), 85.8 (Cc), 79.9 (Cb), 79.5 (Cb), 77.0 (Cd), 18.6 (Ce) and 8.1 (C9).

[3-Methyl-4-oxo-(1*H*-κ*N*^2^-pyrazol-1-yl)-1,4-dihydronaphtalene-1,2-bis(olato)-κ*O*^1^-κ*O*^2^)(*η*^6^-indane)ruthenium(II)](**4**)

The reaction was performed according to the general procedure using [RuCl_2_(indane)]_2_ (150 mg, 0.26 mmol), 1*H*-pyrazole (40 mg, 0.59 mmol), phthiocol (111 mg, 0.59 mmol) and NEt_3_ (259 µL, 188 mg, 1.86 mmol). The mixture was stirred at 50 °C for 20 minutes under microwave irradiation. Flash chromatography with EtOAc/*n*-hexane/NEt_3_ (80/10/2). Yield: 144 mg yellow/green solid (0.30 mmol, 58%). Elemental analysis found: C, 57.58; H, 4.29; N, 6.08; O, 10.50. Calc. for C_23_H_20_N_2_O_3_Ru·(H_2_O)_0.25_: C, 57.79; H, 4.32; N, 5.86; O, 10.88%. ^1^H NMR (600.25 MHz, MeOD-d_4_) δ 8.32 (d, *J* = 2.3 Hz, 1H, H3′), 8.12–8.08 (m, 1H, Harom.), 7.63–7.57 (m, 3H, Harom.), 6.68 (d, *J* = 2.6 Hz, 1H, H5′), 6.34 (dd, *J* = 2.4, 2.4 Hz, 1H, H4′), 5.97 (d, *J* = 5.7 Hz, 1H, Hb/d), 5.93 (d, *J* = 5.8 Hz, 1H, Hb/d), 5.87 (dd, *J* = 5.6, 5.6 Hz, 1H, Ha/c), 5.65 (dd, *J* = 5.5, 5.5 Hz, 1H, Ha/c), 2.99–2.92 (m, 1H, Hf), 2.90–2.84 (m, 1H, Hf), 2.83–2.75 (m, 2H, Hf), 2.17–2.04 (m, 2H, Hg) and 1.75 (s, 3H, H9). ^13^C NMR (150.93 MHz, MeOD-d_4_) δ 183.9 (C4), 183.4 (C2), 141.5 (C3′), 137.8 (C4a/8a), 134.1 (C4a/8a), 132.3 (C5–8), 131.3 (C5–8), 128.0 (C5′), 127.8 (C5–8), 127.1 (C5–8), 108.7 (C4′), 105.7 (C3), 102.4 (Ce), 100.1 (Ce), 94.8 (C1), 82.2 (Ca/c), 80.4 (Ca/c), 79.4 (Cb/d), 78.9 (Cb/d), 30.9 (2xCf), 24.1 (Cg) and 8.1 (C9).

[3-Methyl-4-oxo-(1H-κ*N*^2^-pyrazol-1-yl)-1,4-dihydronaphtalene-1,2-bis(olato)-κ*O*^1^-κ*O*^2^)(*η*^6^-biphenyl)ruthenium(II)](**5**)

The reaction was performed according to the general procedure using [RuCl_2_(biphenyl)]_2_ (100 mg, 0.15 mmol), 1*H*-pyrazole (21 mg, 0.31 mmol), phthiocol (55 mg, 0.29 mmol) and NEt_3_ (128 µL, 93 mg, 0.92 mmol). The mixture was stirred at 50 °C for 20 minutes under microwave irradiation. Flash chromatography with EtOAc/MeCN/NEt_3_ (88/10/2). Yield: 144 mg yellow/green solid (0.19 mmol, 66%). Elemental analysis found: C, 60.76; H, 4.12; N, 5.53; O, 9.86. Calc. for C_26_H_20_N_2_O_3_Ru·(H_2_O)_0.2_: C, 60.86; H, 4.01; N, 5.46; O, 9.98%. ^1^H NMR (600.25 MHz, MeOD-d_4_) δ 8.10–8.07 (m, 1H, H5–8), 7.99 (d, *J* = 2.2 Hz, 1H, H3′), 7.87–7.82 (m, 2H, Hf–h), 7.61–7.57 (m, 3H, H5–8), 7.53–7.49 (m, 3H, Hf–h), 6.66 (d, *J* = 2.5 Hz, 1H, H5′), 6.26 (dd, *J* = 2.4, 2.4 Hz, 1H, H4′), 6.24 (d, *J* = 6.2 Hz, 2H, Hb), 6.20 (dd, *J* = 5.9, 5.9 Hz, 1H, Hc), 6.09 (dd, *J* = 5.9, 5.9 Hz, 1H, Hc), 5.92 (dd, *J* = 5.6, 5.6 Hz, 1H, Hd), 1.67 (s, 3H, H9). ^13^C NMR (150.95 MHz, MeOD-d4) δ 184.0 (C4), 182.6 (C2), 141.3 (C3′), 137.4 (C4a–8a), 136.2 (Ce), 134.0 (C4a–8a), 132.4 (Cf–h), 131.3 (C5–8), 130.9 (C5–8), 130.0 (Cf–h), 129.9 (Cf–h), 128.0 (C5′), 127.7 (C5–8), 127.2 (C5-8), 108.6 (C4′), 105.7 (C3), 99.3 (Ca), 95.0 (C1), 84.1 (Cc), 83.8 (Cc), 81.1 (Cb), 80.9 (Cb), 80.7 (Cd) and 8.1 (C9).

[3-Methyl-4-oxo-(1H-κ*N*^2^-pyrazol-1-yl)-1,4-dihydronaphtalene-1,2-bis(olato)-κ*O*^1^-κ*O*^2^)(*η*^6^-hexamethylbenzene)ruthenium(II)](**6**)

The reaction was performed according to the general procedure using [RuCl_2_(hexamethylbenzene)]_2_ (150 mg, 0.23 mmol), 1*H*-pyrazole (37 mg, 0.54 mmol), phthiocol (102 mg, 0.54 mmol) and NEt_3_ (226 µL, 164 mg, 1.62 mmol). The mixture was stirred at 60 °C for 30 minutes under microwave irradiation. Flash chromatography with EtOAc/*n*-hexane/NEt_3_ (80/15/5). The fractions were combined, evaporated and the orange residue was dissolved in dichloromethane. Crystallization with *n*-hexane provides orange crystals, which were separated and dried in vacuo at 60 °C. Yield: 80 mg orange crystals (0.15 mmol, 27%). Elemental analysis found: C, 59.04; H, 5.39; N, 5.46; O, 9.21. Calc. for C_26_H_28_N_2_O_3_Ru·(CH_2_Cl_2_)_0.15_: C, 59.22; H, 5.38; N, 5.28; O, 9.05%. ^1^H NMR (700.40 MHz, MeOD-d_4_) δ 8.11 (s, 1H, H3′), 8.10–8.07 (m, 1H, H5–8), 7.63–7.53 (m, 3H, H5–8), 6.63 (d, *J* = 2.5 Hz, 1H, H5′), 6.34 (dd, *J* = 2.4, 2.4 Hz, 1H, H3′), 2.30 (s, 18H, Hb), 1.76 (s, 3H, H9). ^13^C NMR (176.13 MHz, MeOD-d_4_) δ 185.4 (C4), 183.5 (C2), 139.1 (C3′), 138.4 (C4a/8a), 134.3 (C4a/8a), 132.0 (C5–8), 131.0 (C5–8), 128.2 (C5′), 128.1 (C5–8), 126.9 (C5–8), 108.6 (C4′), 105.4 (C3), 94.5 (C1) 91.8 (Ca), 16.0 (Cb) and 8.0 (C9).

### 2.2. HPLC-MS Stability Measurements

The 10 mM stock solutions were prepared by dissolving compounds 2–6 in DMSO. 4 µL of stock solution was mixed with 990 µL of PBS buffer (pH 7.4) and 6 µL DMSO to obtain a final concentration of 40 µM. After injection, the chromatogram at 225 nm and the corresponding mass spectra (positive mode) were recorded hourly (4 h) and after 24 h at 20 °C. Peak areas were determined by integrating complex signals of the respective chromatogram. The measurements were performed with an HPLC system (Agilent Technologies, 126 Infinity) equipped with a C18 column (Waters, Atlantis T3 3 μM, 1.0 × 150 mm^2^) coupled to an MS (Bruker, amaZon SL, ESI, positive mode) with a flow rate of 0.2 mL/min at 20 °C and a gradient (5–95%) with Milli-Q water/ACN.

### 2.3. Theoretical Simulations

Optimizations of energetic minima and maxima (transition states) were carried out with the ORCA program suite [26] and the PBEh-3c method [27]. The PBEh-3c method is based on DFT that uses the PBE0 functional, reparametrized with 42% Hartree–Fock exchange, and the def2-mSVP double-zeta basis set. It further accounts for dispersion correction, the basis set superposition error, and includes the ZORA effective core potential for the Ru atom. This method has been shown to be more reliable than most frequently applied DFT protocols, such as B3LYP/6-31G*, and turned out to be most suitable for the purpose of this study [26,27]. A tight convergence was set for the self-consistent field and the DFT grid was set to 4. The conductor-like polarizable continuum model [28] was used to model the solvent with a dielectric constant of 80.4 and a refractive index of 1.33. Due to numerical instabilities and to obtain smoother potentials, Gaussian smearing [29] was applied to the point charge. After every converged optimization, a frequency calculation was carried out to confirm a minimum or transition state on the potential energy landscape.

The structure of the aqua complex was estimated by elongation of the bond between the metal and the weaker bound oxygen (next to the C=C double bond in the dihydronaphtalene unit) and by placing a water molecule close to the Ru atom. The initial structures were pre-optimized at the semi-empirical HF-3c level of theory. The relaxed structure was used as an input for subsequent optimization with PBE3h-3c.

In order to estimate the energy barrier for the aquation process, we carried out a transition state search. The optimization was carried out in ORCA with the settings described above. A frequency calculation with a single imaginary frequency confirmed the found transition state.

### 2.4. Cell Culture

CH1/PA-1 ovarian teratocarcinoma cells (provided by L. R. Kelland, CRC Centre for Cancer Therapeutics, Institute of Cancer Research, Sutton, UK; confirmed by STR profiling as PA-1 ovarian teratocarcinoma cells at Multiplexion, Heidelberg, Germany), SW480 colon carcinoma and A549 non-small cell lung cancer cells (both obtained from the American Type Culture Collection, Manassas, VA, USA) were grown as adherent cultures in 75 cm² culture flasks (CytoOne, Starlab, Hamburg, Germany) by using minimal essential medium (MEM) supplemented with 1 mM sodium pyruvate, 4 mM L-glutamine, 1% (*v*/*v*) nonessential amino acids from 100-fold stock (all purchased from Sigma-Aldrich) and 10% heat-inactivated fetal bovine serum (BioWest, Nuaillé, France). Cells were maintained under standard culture conditions with 5% CO_2_ at 37 °C in a humidified atmosphere.

### 2.5. MTT Assay

The 3-(4,5-dimethylthiazol-2-yl)-2,5-diphenyl-2H-tetrazolium bromide (MTT, Acros Organics, Geel, Belgium) assay was used to detect the cytotoxicity of the compounds after 24 h and 96 h incubation. In brief, cells were harvested from culture flasks and the following cell numbers (each in 100 µL) were seeded into 96-well culture plates (CytoOne, tissue culture treated, Starlab, Hamburg, Germany) for 24 h incubation: 1 × 10^4^ (CH1/PA-1), 1 × 10^4^ (SW480) and 1.5 × 10^4^ (A549) cells per well; and for 96 h incubation: 1 × 10^3^ (CH1/PA-1), 2 × 10^3^ (SW480) and 3 × 10^3^ (A549) cells per well. After 24 h, cells resuming their exponential growth rate were treated with test compounds at nine concentrations obtained by serial two-fold dilution. Stock solutions were prepared in DMSO (20 mM or 40 mM) and diluted in supplemented MEM. An amount of 100 µL of each concentration was added to the plates in triplicates and cells were exposed for 24 or 96 h. Afterward, the medium was replaced with 100 µL of MTT solution (5 mg/mL) in phosphate-buffered saline (PBS, Sigma-Aldrich), diluted 1:7 in RPMI 1640 medium (supplemented with 4 mM L-glutamine and 10% heat-inactivated FBS). Upon cell-viability-dependent conversion of the yellow, water-soluble tetrazolium salt into purple formazan products for 4 h, the crystals were dissolved in 150 µL DMSO. The absorbance of the resulting solutions, which is directly proportional to the number of viable cells, was measured at 550 nm (and at 690 nm as a reference) with a microplate reader (ELx808, BioTek, Winoosky, VT, USA) and Gen5TM 3.08 software (BioTek). Blank-corrected optical densities were subjected to graphical evaluation and interpolation of IC_50_ values. At least three biologically and technically independent experiments were performed.

### 2.6. Colony Formation Assay

For the colony formation assay, SW480 cells were seeded at a density of 350 cells/2 mL into each well of 6-well plates (CytoOne, tissue culture treated) and left to adhere for 24 h. To determine the long-term effects of the test compounds, the cells were exposed to them for 11 days at 37 °C and 5% CO_2_. After incubation, the wells were gently washed with 1 mL cold PBS and the colonies were fixed with 1 mL cold (4 °C) 100% methanol (Thermo Fisher, Waltham, MA, USA) for 30 min at 4 °C and washed with 1 mL PBS again. The fixed colonies were stained with 1 mL of 0.5% *w*/*v* crystal violet (Sigma-Aldrich) in methanol for 2 min and the plates were washed under tap water. After drying the plates, images of the wells were captured, and colonies were counted with the colony counting function of Fusion Fx7 software (Vilber Lourmat, Collégien, France). Results were normalized to untreated controls. Data are averaged from at least three independent experiments.

### 2.7. Cellular Ruthenium Accumulation

SW480 cells were seeded at densities of 1.8 × 10^5^ cells/well in 12-well plates (CytoOne, tissue culture treated) in 1 mL of supplemented MEM per well. (As CH1/PA-1 cells tend to detach during the required washing steps, cellular accumulation studies were performed only in SW480 cells). After 24 h, compounds dissolved in DMSO and diluted in MEM were added to the cells to final concentrations of 2 µM and 5 µM, and cells were incubated at 37 °C for 2 h. During incubation, the cell number was determined from three control wells of a plate seeded in parallel to the treatment plates. After the 2 h incubation, the medium was removed and all wells were washed with 3 × 1 mL PBS, and cells were lysed by adding 400 µL HNO_3_ per well for 1 h at room temperature. An amount of 300 µL of each lysate was added to 7.7 mL MilliQ water to get a total volume of 8 mL, and determination of the ruthenium content was carried out by ICP-MS. The measured amount of accumulated metal was normalized to the cell number and results were given in fg [Ru]/cell from at least three independent experiments. An Agilent 7800 ICP-MS instrument (Agilent Technologies, Tokyo, Japan) equipped with an Agilent SPS 4 autosampler (Agilent Technologies, Tokyo, Japan) was used to determine the ruthenium content. The ICP-MS parameters were tuned on a daily base to achieve high sensitivity. The ICP-MS was equipped with a MicroMist nebulizer with a sample uptake rate of ∼0.2 mL/min and standard nickel cones. The Agilent MassHunter software package (Workstation Software, Version C.01.04, 2018) was used for data evaluation. The instrumental parameters can be found in the supplementary information (Table S9).

### 2.8. Cell Cycle Studies

For the flow-cytometric determination of cell cycle distribution, CH1/PA-1 (8 × 10^4^ cells/well) and SW480 (1.2 × 10^5^ cells/well) cells were seeded into 12-well plates (CytoOne, tissue culture treated) in 1 mL supplemented MEM. In the first 24 h, the cells were allowed to settle and resume exponential growth. Afterward, the cells were treated with three different concentrations of the test compounds (~half of IC_50_, ~IC_50_ and ~two times IC_50_) and continuously exposed for 24 h at 37 °C and at 5% CO_2_. Gemcitabine (Sigma-Aldrich, 0.01 and 0.05 µM) and etoposide (Sigma-Aldrich, 0.25, 0.5 and 1 µM) were used as positive controls. After removal of the treatment medium, cells were washed with 1 mL PBS and trypsinized. Cells were collected in Eppendorf tubes and centrifuged (300 g, 3 min), and the supernatant was discarded. In the following step, cells were washed with 1 mL PBS and resuspended in 500 µL PI/HFS buffer (0.1% *v*/*v* Triton X-100, 0.1% *w*/*v* sodium citrate, in PBS) containing 25 µg/mL propidium iodide (PI, 1.0 mg/mL, Sigma-Aldrich). The staining was carried out overnight at 4 °C in the dark, and 200 µL of the cell suspension was loaded into 96-well round bottom plates (Falcon). Samples were measured with a Millipore Guava easyCyte 8HT flow cytometer with GuavaSoft™ software in the InCyte-modus, and 10,000 events/sample were detected. Data were analyzed with FlowJo software v 10.8.1 (Tree Star, Ashland, OR, USA) using Watson algorithm. Percentages of cells in the three cell cycle phases (G1/G0, S and G2/M) were calculated by integrating the resulting histograms of fluorescence signals (Figures S29 and S30). Experiments were repeated at least three times. For statistical evaluation, an unpaired *t*-test with Welch’s correction was performed by using GraphPad PRISM software.

### 2.9. Apoptosis Assay (Annexin V/PI)

Cell death induction was quantitatively analyzed via flow cytometry using double staining with FITC-conjugated annexin V (eBioscience, San Diego, CA, USA) and propidium iodide (PI, 1.0 mg/mL, Sigma-Aldrich). SW480 cells were seeded into 24-well plates (7 × 10^4^ cells/well) in 600 µL MEM per well and allowed to settle for 24 h. Afterward, cells were exposed to two or three different concentrations (0.1–100 µM) of the compounds (based on their cytotoxic activity) for 24 h. After treatment, the medium was collected, and cells were washed once with 37 °C warm PBS and harvested. Following trypsinization, the cell suspension was added to the pre-collected medium and cells were centrifuged (300 g, 3 min). The supernatant was removed, and the cell pellet was resuspended with 1.5 µL FITC-conjugated annexin V in 150 µL binding buffer (10 mM HEPES/NaOH pH 7.4, 140 mM NaCl and 2.5 mM CaCl_2_) and incubated at 37 °C for 15 min. An amount of 1 µL of propidium iodide (PI, Thermo Fisher) was added shortly before flow-cytometric analysis using a Guava easyCyte 8HT instrument (Merck Millipore, Burlington, MA, USA) with InCyte software. Results were quantified by using the FlowJo software (TreeStar). At least three independent experiments were conducted.

### 2.10. DCFH/DA Assay

To monitor the cellular levels of reactive oxygen species (ROS), SW480 cells were seeded into 96-well plates (2.5 × 10^4^ cells/well) in volumes of 100 µL per well. After a 24 h pre-incubation, the cells were washed with 200 µL Hanks’ balanced salt solution (HBSS, Sigma-Aldrich, containing 1% FCS) and incubated with 100 µL of 25 µM DCFH-DA (2′,7′-dichlorofluorescein diacetate, Sigma-Aldrich) in HBSS (containing 1% FCS) for 45 min at 37 °C. Cells were washed with 200 µL HBSS and exposed to the compounds in a range of concentrations (0.2–200 µM) in 200 µL phenol-red-free Opti-MEM (Gibco, with 1% FCS) in triplicates. All experiments included blanks (phenol-red-free medium with 1% FCS), negative controls (non-drug-treated cells) and positive controls (cells treated with 200 µM and 400 µM tert-butyl hydroperoxide (Sigma Aldrich)). ROS production was measured at 10 min intervals with the Biotek Synergy HT reader (excitation: 485/20 nm, emission: 516/20 nm) over a 2 h period. For representation, the values were given as the ratios of the means of treated samples and the means of the negative control ± standard deviations from two independent experiments (due to a lack of effect, no more repeats were performed).

### 2.11. DNA Interaction Assay

For detecting changes in DNA secondary structure upon drug treatment, 400 ng of the pUC19 plasmid (2686 bp, New England Biolabs, Ipswich, MA, USA) was incubated with the test compounds at a final concentration of 50 µM (dissolved and diluted in MilliQ water). The incubation was performed at 37 °C for different time intervals (15 min to 6 h) with continuous shaking. Additionally, 4 µL aliquots of a 6× DNA loading dye (Thermo Fisher) were added to the 20 µL reaction solution, and the DNA samples were separated in a 1% agarose gel in 1 × Tris-Borate-EDTA (TBE) buffer. Electrophoresis was initiated at 60 V for 5 min and continued at 120 V for 90 min in 1 × TBE buffer. For visualization of the DNA modification degree, the agarose gel was stained with ethidium bromide (EtBr) in 1 × TBE (0.75 µg/mL) for 20 min. Images were captured with the GelDoc-It Imaging System Fusion Fx7 (Vilber Lourmat, Eberhardzell, Germany), and data were quantified with ImageJ/Fiji 1.46 software.

### 2.12. NQO1 Activity Assay

To determine NQO1 activity, SW480 and CH1/PA-1 cells were seeded into 6-well plates in densities of 2 × 10^5^ cells/well in 2 mL supplemented MEM. After the recovery time of 24 h, cells were treated with two or three different concentrations of test compounds (0.1–100 µM, based on the corresponding IC_50_ values). The test substances were dissolved in DMSO/medium. To exclude that assay validity was compromised by the use of DMSO, cells treated with DMSO alone (at concentrations corresponding to those in substance solutions) were measured in parallel. After 24 h drug exposure, the medium was aspirated, and the cells were gently washed twice with PBS. The further steps were performed following the manual instructions of the NQO1 activity assay kit (ab184867, abcam, Cambridge, UK). Protease inhibitor phenylmethylsulfonyl fluoride (PMSF, Merck, Darmstadt, Germany) and phosphatase inhibitor PhosSTOP^TM^ (Roche, Basel, Switzerland) were applied. For determining the protein concentration, the Micro BCA^TM^ protein assay (prod # 23235, Thermo Scientific) kit was used. For NQO1 activity determination, 100 µg/mL of total protein was loaded. The development of yellow color was monitored for 5 min in 20-sec intervals with a microplate reader (BioTek ELx808) at 450 nm. The measured data were evaluated according to the instructions of the NQO1 activity assay kit: “NQO1 activity was calculated by subtracting the OD value with Inhibitor from the one without Inhibitor. The activity is expressed as the change in absorbance per minute per amount of sample loaded into the well” [30]. Data are expressed as rates of increase of mean absorbance ± SDs from three independent experiments.

## 3. Results and Discussion

### 3.1. Syntheses and Characterization

**Scheme 1:**
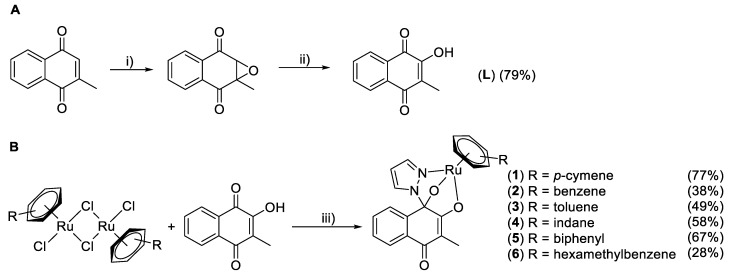
Synthetic pathway of phthiocol (**L**) (**A**) and complex syntheses (**1**–**6**) (**B**). (i) = H_2_O_2_, Na_2_CO_3_, MeOH; 0 °C to room temperature, 1.5 h; (ii) = SiO_2_, H_2_SO_4_, THF, 70 °C; (iii) = MeOH, microwave, 50–60 °C, 20–30 min.

The dimeric ruthenium arene precursors were synthesized according to the literature’s procedures or purchased from commercial suppliers ([Ru(η^6^-hexamethylbenzene)Cl_2_]_2_) [21,22,23]. The desired dienes were obtained via Birch reduction (2,3,4,7-tetrahydro-1*H*-indene and 1,4-dihydro-1,1′-biphenyl) or were commercially available (α-terpinene, 1,4-dihydrocyclohexen and 1-methylcyclohexa-1,4-diene) [31,32]. Treatment of the respective diene with RuCl_3_ × H_2_O yielded the desired dimeric precursors in good to excellent yields (71–98%). 2-Hydroxy-3-methyl-1,4-naphthoquinone (phthiocol, **L**) was synthesized according to the literature, where menadione was formed via epoxidation and subsequent SiO_2_-mediated ring opening (yield: 79%) [24,25]. The tridentate complexes were obtained by treating the respective ruthenium dimer, 1*H*-pyrazole and an excess of triethylamine in methanol under microwave irradiation, resulting in moderate to good yields (28–77%) (Scheme 1) [17,19]. The enhanced complex stabilities allowed a straight-forward purification by column chromatography with a ternary eluent system. Furthermore, this complex class exhibits two stereo centers, one at the C1 atom, which connects the pyrazole moiety with the phthiocol ligand, and one at the Ru(II) itself. Theoretically, four stereoisomers should occur, however, only two isomers can be formed due to geometrical reasons (*R*_C1_, *R*_Ru_ and *S*_C1_, *S*_Ru_) [17].

The low yield of **6** may result from the favored formation of the parental chlorido complex, which could be isolated via column chromatography during the work up. However, the bidentate chlorido compound was not investigated in this project, due to the focus on tridentate complexes.

**Figure 3:**
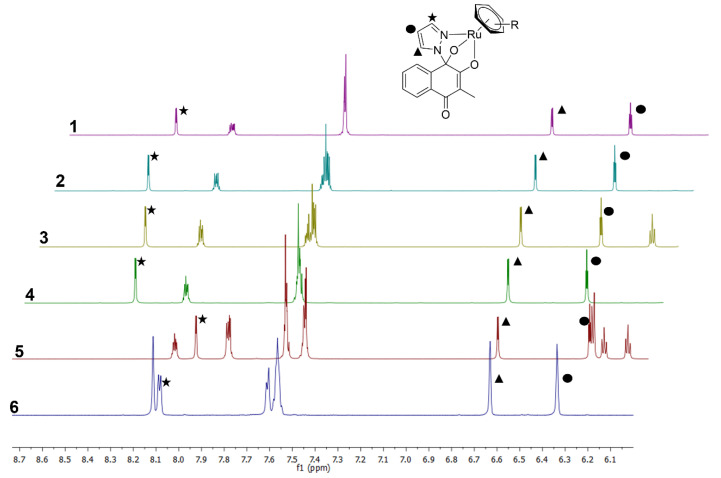
Arene influence towards the chemical shifts of pyrazolate in MeOD-d_4_. R = *p*-cymene = **1**, R = benzene = **2**, R = toluene = **3**, R = indane = **4**, R = biphenyl = **5**, R = hexamethylbenzene = **6**.

All complexes were characterized with standard analytical methods and the purity was proved by using elemental analysis. In the ^1^H-NMR spectra of **1**–**6**, the signal corresponding to the pyrazolate proton in position three (denoted by an asterisk in Figure 3) was shifted the most upon complex formation due to the spatial proximity to the arene. The electron-rich arenes shift the signal to higher fields. The other pyrazolate protons and those of the naphthoquinone showed only negligible shift changes, due to the increased distance to the arene. The occurrence of a quaternary carbon signal around 90–100 ppm confirmed the formation of a hemiaminal bond (Figures S1–S10).

### 3.2. X-ray Crystallographic Studies

**Figure 4:**
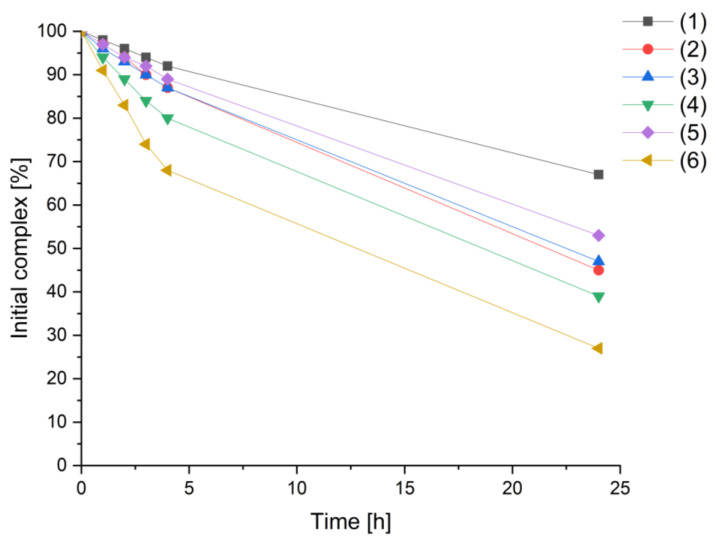
The percentage amount of initial complex vs. time in PBS (pH = 7.4) at 20 °C.

Single crystals of four compounds (**2**–**4**, **6**) were obtained by vapor diffusion or the liquid–liquid diffusion method from dichloromethane/diethyl ether or dichloromethane/*n*-hexane. The respective ORTEP plots and sample data can be found in the supplementary information (Figures S11–S14 and Tables S1–S5). The toluene derivative (**3**) crystallized in the orthorhombic space group *Ibca*, compound **4** in the triclinic group *P*-1 and the other complexes in monoclinic space groups (*C*2/*c* for **1**, *P*2_1_/*n* for **2** and **6**). For all complexes, the following order of bond lengths was observed: Ru-*O*2 > Ru-*N*2 > Ru-*O*1. It confirmed the hydrolysis mechanism reported in our previous work, where the Ru-*O*2 bond cleaved firstly to obtain the aqua complex [19]. A comparison of the bond lengths and the stability data showed no correlation (Table 1, Figure 4).

### 3.3. Stability Studies

Complex stability in an aqueous solution is an important parameter for drug candidates since the intact compound should reach the tumor tissue in sufficient amounts to take effect [33]. Transition metal complexes tend to hydrolyze in an aqueous solution, yielding highly reactive aqua species which can rapidly interact with biomolecules. Therefore, the aqueous behavior of all complexes (**1**–**6**) under physiologically relevant pH (7.4) at 20 °C was investigated via HPLC-MS. The percentage amount of the initial complex was determined by the integration of the complex signal hourly (for 4 h) and after 24 h (Figure 4).

The complex signals were detected between 12.1–15.9 min with the corresponding masses [M+H]^+^ and [M−pz]^+^ (Figures S15–S20). Depending on the arene, hydroxido- and pyrazolato-bridged dimers were observed in a time-dependent manner.

The *p*-cymene complex (**1**) showed the highest stability, with more than 70% of the original complex still existing after 24 h. Similar aqueous behavior was observed for compounds **2**–**5**, with 40–60% of the initial complexes persisting. The hexamethylbenzene derivative (**6**) exhibited the lowest stability, with less than 70% of the complex remaining intact after 4 h. The reduced stability, poor yield and formation of the parental bidentate chlorido complex suggest that tridentate pyrazolate naphthoquinone complex **6** exhibits an unfavorable structure.

### 3.4. DFT Calculations

**Figure 5:**
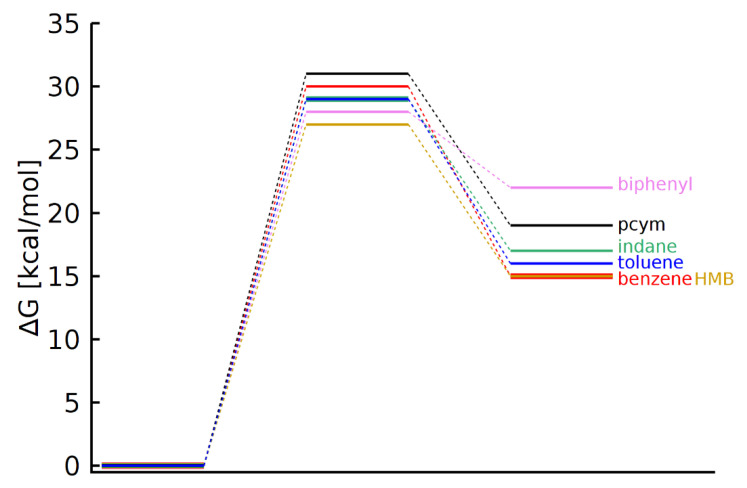
DFT calculations regarding the hydrolysis of complexes (**1**–**6**). Relative free energies of complexes (**1**–**6**) are set to zero (left) in order to compare the energetic order of the corresponding transition states (middle) and aqua complexes (right).

In order to obtain further insight into the complexes’ stabilities, DFT calculations of all complexes (**1**–**6**), their corresponding hydrolyzed aqua species, and the respective transition states were carried out (Table 2). The energies of complexes (**1**–**6**) are set to zero for better comparison of barrier heights (Figure 5). According to the results, the experimentally found stability (Figure 4) can only be explained by a complex interplay of follow-up reactions, kinetic effects and thermodynamics.

The first striking point is that the complexes (**1**–**6**) should be more stable than their hydrolyzed counterparts. The experimentally found decomposition is likely due to follow-up reactions, where, e.g., the aqua complexes dimerize and, in this way, shift the equilibrium of the hydrolyzation reaction towards the aqua products. Furthermore, the relative stability of the complexes (**1**–**6**) can neither be explained alone by the energetic order of the product energies (aqua complexes) nor alone by the energetic order of the respective transition states. Instead, an interplay of transition barrier heights and product energy (i.e., a combination of kinetic and thermodynamic effects) leads to the experimentally found stabilities. This complex interplay obstructs the rational design of more stable complexes because the hydrolyzation stability cannot be predicted with a simple rule of thumb.

### 3.5. Biological Studies

#### 3.5.1. Effects on Cell Proliferation and Cytotoxicity

The cytotoxicity of compounds **1**–**6** was determined in three different cancer cell lines after 24 h and 96 h incubation (Figures S21 and S22). These compounds exhibit a similar cytotoxic profile to those reported previously, where low activity was observed in CH1/PA-1 and high activity in A549 and SW480 cancer cells [17,19]. The IC_50_ values in CH1/PA-1 cells of the complexes (**1**–**6**) were only slightly lower or in the same range as that of phthiocol (**L**) (Table 3). In contrast, the data indicate a pronounced selectivity for SW480 cells. In the latter, the lowest IC_50_ values were observed for compounds **1** and **5** (bearing an electron-rich arene) after 24 h as well as after 96 h incubation.

These observations lead to the assumption that lipophilicity plays a major role in the activity profile, with more lipophilic compounds showing higher cytotoxicity. Plotting the IC_50_ values (Table 3) against the calculated miLogP values of the different arenes (www.molinspiration.com, Table S6, (accessed on 13 October 2022) showed a good agreement between cytotoxicity and calculated lipophilicity (Figure S23), demonstrating that the more lipophilic compounds **1**, **5** and **6** had the lowest IC_50_ values. The benzene and toluene derivatives (**2**, **3**), on the other hand, exhibited the lowest cytotoxicity in all treated cancer cell lines. Complex **4**, with indane as the arene, is an exception since it has similar miLogP and stability values to **2** and **3** but an enhanced cytotoxicity in all three cancer cells.

**Figure 6:**
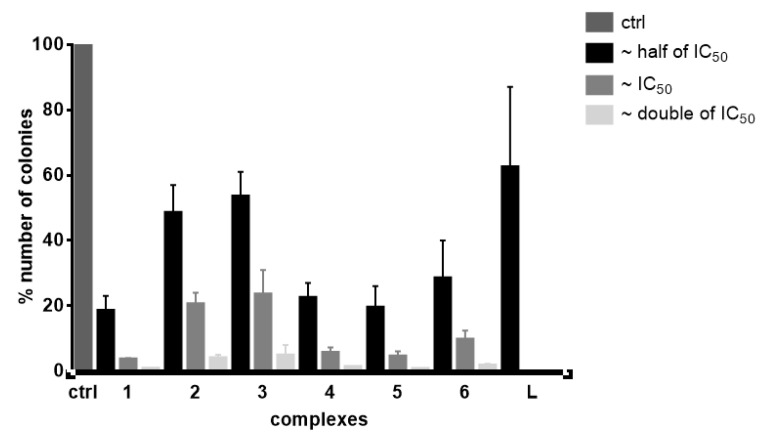
Inhibition of colony formation in SW480 cells exposed to **L** and **1**–**6** at two or three different concentrations for 11 days. The results are given in percentage (%) relative to the untreated control. Columns represent the mean ± standard deviation of three independent experiments.

To determine the long-term effect of drugs on proliferating tumor cells, a colony formation assay (CFA) was performed for 11 days [37]. The ability of single cells to grow into a colony was tested at three different concentrations based on IC_50_ values of 96 h MTT results in SW480 cells (Figures S24–S27 and Figure 6). After 11 days of exposure, only a minor fraction of seeded cells retained the capacity for growth at a concentration of about half of the 96-h IC_50_ values. For the two most cytotoxic compounds, **1** and **5**, 18 ± 5% and 19 ± 7% colonies, respectively, were counted (relative to the untreated control) (Table S7). The determination of clonogenic growth at a concentration of about the 96-h IC_50_ value yielded the expected results: 3.1 ± 0.8% (compound **1**) and 4.0 ± 2.0% (compound **5**), which correlates well with the results of the MTT assay (24 and 96 h). Treatment with a concentration of approximately the double 96-h IC_50_ value significantly decreased the colony formation upon treatment with all substances, as almost no colonies were observed. Remarkably, the phthiocol ligand (**L**) showed strong inhibition of colony growth (at the 96-h IC_50_ no colonies could be detected), even though it showed modest cytotoxicity (116 ± 37 µM) after 96 h treatment in the MTT assay.

#### 3.5.2. Cellular Accumulation

**Figure 7:**
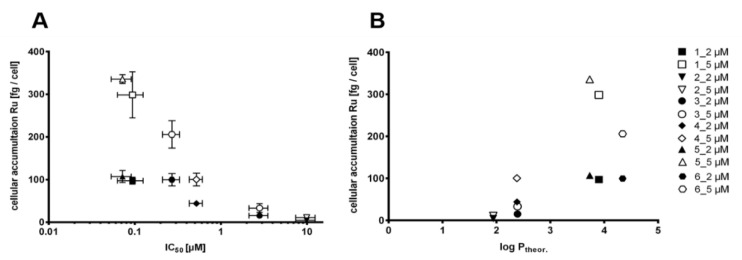
Semi-logarithmic plot of cellular accumulation after 2 h exposure to compounds **1**–**6** vs. their IC_50_ values (96 h exposure) in SW480 cells (**A**). Correlation between drug accumulation and a theoretical lipophilicity coefficient (96 h exposure) (**B**). The symbols indicate the compound concentrations applied in cellular accumulation experiments, with filled symbols for 2 µM and open symbols for 5 µM.

In order to understand if there is a correlation between cytotoxicity, cellular accumulation and lipophilicity, the total cellular ruthenium content was quantified by ICP-MS. After 2 h of incubation with the compounds **1**–**6**, SW480 cells were lysed and analytically diluted for metal quantification. The diversity in structure among the (arene) ligands is reflected in a wide range of cellular accumulation of ruthenium from 4.5 fg to 107 fg per cell (treated with 2 µM of the test compound) and from 11 fg to 336 fg per cell (treated with 5 µM of the test compound, Table S8). According to the quantified ruthenium content, the ligands can be ranked with regard to cellular accumulation of the corresponding complex as follows: benzene (**2**) < toluene (**3**) < indane (**4**) < hexamethylbenzene (**6**) < *p*-cymene (**1**) < biphenyl (**5**) (Figure S28). The highest cellular ruthenium levels were observed for the most cytotoxic compounds (**1**, **5** and **6**). Complex **4**, with milder cytotoxicity, yielded a lower level of cellular accumulation, as expected. The ruthenium accumulation was relatively limited in complexes **2** and **3**, as only minor amounts of the metal were detected, suggesting that the coordinated arene may be unfavorable for membrane permeation. Overall, drug accumulation correlates fairly well with the cytotoxicity and lipophilicity of the complexes (Figure 7).

#### 3.5.3. Analysis of Cell Cycle Distribution

To deepen the understanding of how the novel naphthoquinone complexes exert their biological activity, cell cycle distribution was studied in CH1/PA-1 and SW480 cells upon treatment with these compounds (Figures S29 and S30). In several publications, a significant impact on the cell cycle was reported for naphthoquinone derivatives, which can target the checkpoints of the cell cycle and thereby affect cell proliferation [14,38,39]. In this approach, percentages of cells in G1/G0 (cells preparing for DNA replication/quiescent state), S (cells performing DNA synthesis) and G2/M (cells preparing for/undergoing mitosis) phases can be detected by flow cytometry. In both cell lines, three different concentrations (based on IC_50_ values) were applied to cover a wider range for the detection upon 24 h treatment.

In the CH1/PA-1 cell line, a characteristic pattern could be observed for all tested complexes (Figure S31 and Table S10). The G1/G0 phase fraction is reduced by complexes **1**–**6** at all concentrations tested, whereas the S phase fraction remains unchanged, or is slightly increased by complex **1** (up to 11% relative to untreated control) in a concentration-dependent manner. An increase in the cell fraction in the G2/M phase relative to the control was measured in all cases. The effect of the ligand (**L**) on cell cycle distribution deviates from the trend observed for complexes **1**–**6** in CH1/PA1 cells. The ligand caused a gradual increase in the G1/G0 cell fraction and a decrease in the G2/M fraction, while the S phase was unaffected.

**Figure 8:**
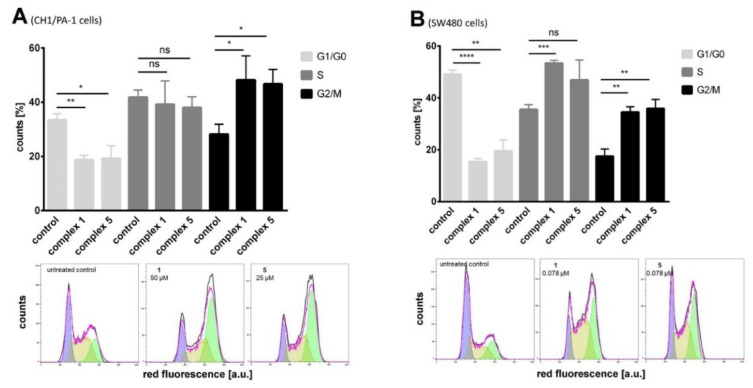
Impact of the most cytotoxic complexes **1** and **5** on cell cycle distribution in CH1/PA-1 (**A**) and SW480 cells (**B**) upon exposure for 24 h. Complex **1** was applied at 50 µM concentration in CH1/PA-1 cells and 0.078 µM in SW480 cells. Complex **5** was applied at 25 µM in CH1/PA-1 cells and 0.078 µM in SW480 cells. Histograms represent the effects of the IC_50_ (according to MTT assays) relative to untreated control in a representative experiment. The different colors indicate the cell fractions in G1/G0 phase (purple), S phase (yellow) and G2/M phase (green), calculated by integrating the histograms of fluorescence signals by using Watson algorithm (indicated by magenta lines). In the column diagrams, the data are given as means ± SDs from at least three independent experiments. Unpaired *t*-test with Welch’s correction was performed for statistical evaluation (* *p* < 0.05; ** *p* < 0.01; *** *p* < 0.001; **** *p* < 0.0001; ns: not significant).

In the SW480 cell line (which is more sensitive to complexes **1**–**6**), the cell cycle distribution was affected more strongly than in the CH1/PA-1 cell line (Figure S32 and Table S11). All compounds induced a significant reduction of cell number in the G1/G0 phase and correspondingly increased the cell fraction in S and G2/M phases. In general, a G2/M phase arrest (up to 28% increase relative to untreated control) or S phase arrest (up to 22% increase relative to the untreated control) could be observed. The ligand (**L**) did not show any cell cycle perturbation in SW480 cells at tested concentrations. In conclusion, the investigated complexes have effects on DNA replication and cell division. The most pronounced effect on the cell cycle was measured for complexes **1** and **5** at concentrations around the respective IC_50_ values in both cell lines (Figure 8).

#### 3.5.4. Apoptosis Induction

Regarding the types of cell death that are induced by anticancer compounds, two main categories can be distinguished: necrosis and programmed cell death, with the latter comprising apoptosis and non-apoptotic mechanisms (such as autophagy, necroptosis, and apoptosis-like programmed cell death) [40,41]. In this study, the ability of apoptosis and necrosis induction was examined by the flow-cytometric annexin V/propidium iodide (AV/PI) assay. The double staining applied in this assay enables the differentiation of viable cells (AV-/PI-), early apoptosis (AV+/PI-), late apoptosis (AV+/PI+) and necrosis (AV-/PI+). If cancer cells undergo early or late apoptosis, phosphatidylserines (PS) are located from the inner leaflet to the outer side of the lipid bilayer of the plasma membrane and (FITC-coupled) annexin V can selectively bind to PS. At the same time, PI can be used for the identification of cells with damaged cell membranes such as necrotic or late apoptotic cells.

**Figure 9:**
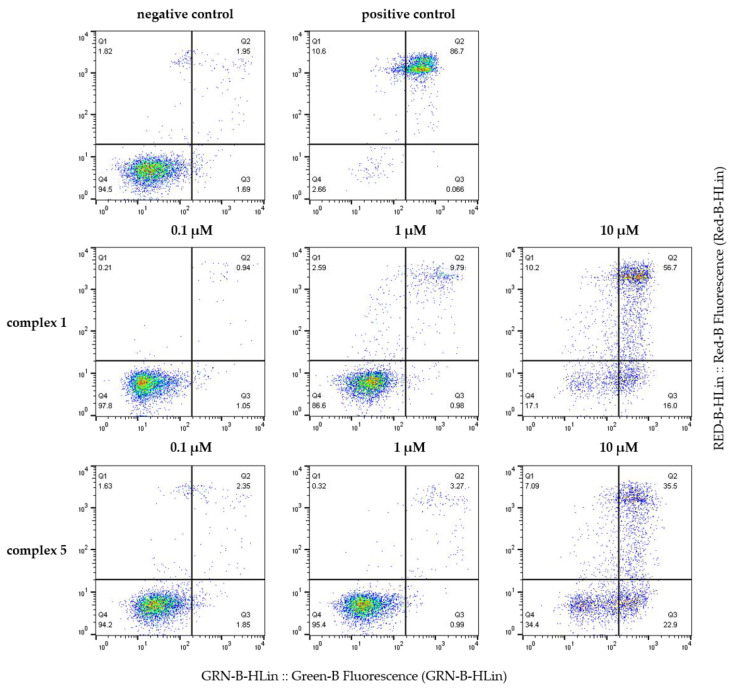
To distinguish the viable (Q4), early apoptotic (Q3), late apoptotic (Q2) and necrotic (Q1) cell counts, SW480 cells treated with complexes **1** and **5** at three different concentrations were subject to annexin V-FITC (x-axis) and PI (y-axis) staining, detected by flow cytometry. As a positive control, 1-P was used [42]. The dot plots represent a representative experiment out of at least three.

The SW480 cells were treated with complexes **1**–**6** and free ligand **L** at concentrations that roughly cover the wide IC_50_ range (24 h incubation) (Table S12, Figure S33). Only complexes **1** and **5** induced apoptosis to a high extent, which increased with concentration (Figure 9). With the highest applied concentration (10 µM) they had a strong influence on the percentages of viable (AV-/PI-), early apoptotic (AV+/PI-) and late apoptotic (AV+/PI+) cells. The increase of late apoptotic cells to 64 ± 6% (complex **1**) and 40 ± 6% (complex **5**) is very prominent. In accordance with the data above, apoptotic events were expected of complex **6** carrying a hexamethylbenzene ligand, but neither apoptotic nor necrotic cells exceeded the percentages in untreated controls. The other tested complexes **2**–**4** as well as **L** did not induce any of these processes either.

#### 3.5.5. ROS Assay

Naphthoquinones are obvious candidates for testing the capacities of reactive oxygen species (ROS) production due to their ability to easily accept electrons, resulting in the formation of semi-quinones/hydroquinones on the one hand, and radical formation of an electron–donor substrate on the other hand. Those radical species undergo reactions rapidly, thereby causing damage such as DNA mutations, which cells might not be able to repair properly. Alternatively, ROS are able to inactivate biologically important enzymes, e.g., ubiquitin-specific proteases 1 and 2 (USP 1 and USP 2), which are associated with the repair of DNA damage and resistance to apoptosis induction [14].

To check their ability to induce ROS in cancer cells, the DCFH-DA-assay has been applied to the new naphthoquinone-comprising metallodrugs (complexes **1**–**6**) and their parental ligand (**L**). The 2′,7′-dichlorodihydrofluorescein diacetate (DCFH-DA) precursor is oxidized by reactive oxygen species, and the product DCF emits fluorescence signals, which may increase or decrease in a time-dependent manner. As compounds are most cytotoxic in SW480 and less active in CH1/PA-1 cells, both cell lines were treated with the compounds. Results indicate no noteworthy increase in ROS levels for all tested complexes or the parental naphthoquinone ligand (Figures S34 and S35). At higher applied concentrations (200 µM for CH1/PA-1 and 20 µM for SW480) slightly reduced levels of ROS could be detected compared to the negative control, while complex **1** even caused overall lowered ROS levels (approximately half the amount of the negative control: T/C ≈ 0.5) with a further decrease at the highest concentration in CH1/PA-1 cells. Intriguingly, complex **5** seems to be the most constant compound, with T/C-values around 0.9–1 throughout the concentration range. Therefore, increased amounts of reactive oxygen species do not seem to be responsible for the cytotoxic potency of the complexes.

#### 3.5.6. DNA Interaction Studies

The compounds’ capacities to alter DNA secondary structure were investigated in a cell-free environment with a dsDNA assay to assess whether DNA could be a possible target of their mode of action. For the assay, intact pUC19 plasmid (2.7 kbp) was incubated with complexes (**2**–**6**) for up to 6 h, and the possible changes in plasmid secondary structure were detected by agarose gel electrophoresis. The plasmid dsDNA mostly holds a negatively supercoiled (sc) form, and may, upon drug treatment, gradually converge to the open circular (oc) form. For the compounds studied here, the only signs of interaction detected were single-strand breaks induced by a few of them (Figure S36). However, other forms of DNA interaction such as cross-linking, intercalation, or double-strand breakage could not be observed, in contrast to effects reported for the ruthenium(III) complexes KP1019 and KP418, which may cause untwisting and bending of DNA [43]. Compounds **2**–**4** moderately induced single-strand breaks; after 6 h of incubation the oc form reached 11.8 ± 0.9%, 11.7 ± 1.6% and 12.1 ± 1.9%, respectively (Figure S37). The interaction showed a clear time dependency, with the detected amount of oc form increasing over time in comparison to the baseline value of 3.6 ± 0.7% (control after 6 h). These findings are nevertheless remarkable because the other compounds (**1**, **5**, **6** and **L**) demonstrated only a minor influence on pUC19 [19]. Apparently, DNA interaction may not contribute to the cytotoxic behavior of the tested complexes, as **2**–**4** only exhibited moderate cytotoxicity in comparison to the other test substances. Overall, this supports the hypothesis that this series of complexes do not have a platinum-like mode of action (*cf.* cisplatin and related platinum drugs) [19,44].

#### 3.5.7. NQO1 Activity

In the literature, it has been reported that naturally occurring naphthoquinones, such as β-lapachone, act as effective cytotoxic agents and selectively kill cancer cells with high expression levels of the NQO1 enzyme (located primarily in the cytosol) [45]. Furthermore, NQO1 activity has been reviewed by Pereyra et al. as a potential mechanism of how naphthoquinones eliminate cancer cells [14]. NAD(P)H:quinone oxidoreductase 1 (NQO1, E.C.1.6.99.2) is a quinone-detoxifying enzyme in normal cells, which utilizes NADH or NADPH as electron donors for the two-electron reduction of quinones to unstable, reactive hydroquinones. The enzyme protects the structure and functioning of normal cells against quinones (exogenous and/or endogenous) and prevents tumor progression. Inhibiting its activity in cancer cells may support therapeutic efficacy by suppressing cancer cell growth or by potentiating the activity of chemotherapeutic drugs [46,47]. One of the most studied and widely used inhibitors of the NQO1 enzyme is dicoumarol, which suppresses the enzymatic activity by competing with NAD(P)H [48].

**Figure 10:**
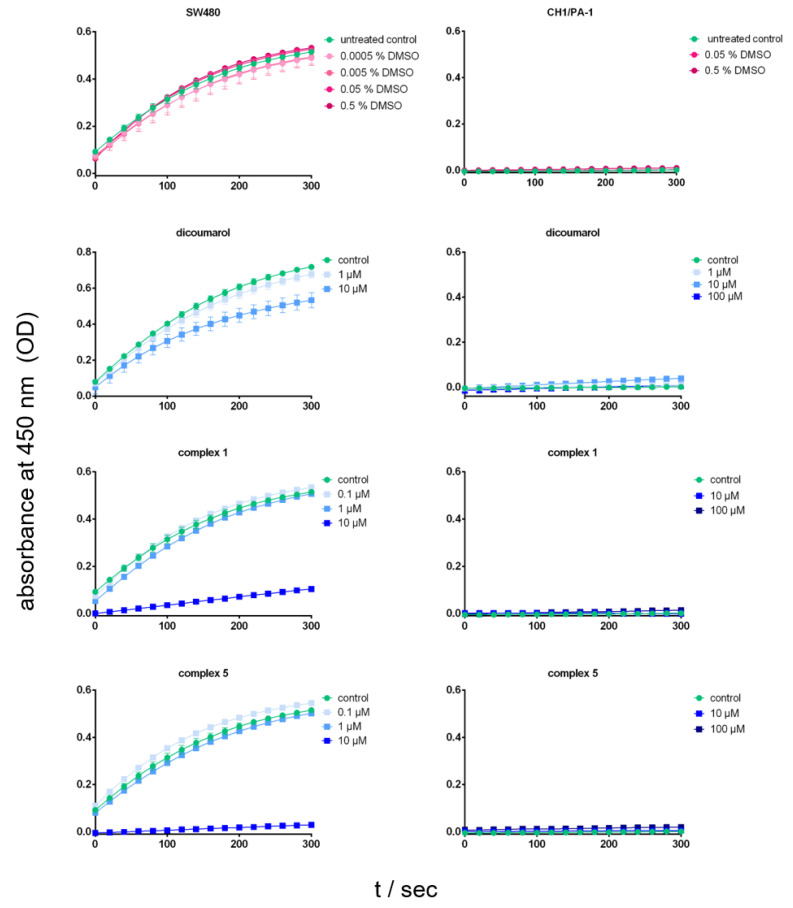
Time-dependent impact on NQO1 activity in SW480 (**left** panel) and CH1/PA-1 (**right** panel) cell extracts after treatment with the most active complexes, **1** and **5**, or dicoumarol (positive control) compared to negative controls with and without DMSO (concentrations corresponding to the content in solutions of the test compounds). Data are expressed as OD (450 nm) per 100 µg/mL of total cellular protein.

Compounds **L** and **1**–**6** are assumed to be substrates of the NQO1 enzyme. They may induce structural changes in NQO1, resulting in the inhibition of the enzyme’s activity in vitro. In SW480 colon carcinoma cells, complexes **1** and **5** lowered NQO1 activity tremendously at 10 µM concentration, which is 21- and 13-fold higher than the corresponding IC_50_ values (cytotoxicity) after 24 h of incubation (Figure 10). At lower concentrations (0.1 µM and 1 µM), negligible effects were detected compared to the untreated control. Compound **4** exhibited a moderate suppression of enzyme activity, also at 10 µM (2.8-fold higher than IC_50_), which may explain its enhanced cytotoxicity compared to **2** and **3** with similar miLogP values and stability. Phthiocol (**L**) at 10 µM moderately inhibited the activity of NQO1 to a similar extent as compound **4** (Figure S38). Experiments were also performed in CH1/PA-1 cells to better understand the unexpected chemoresistance of the usually very sensitive ovarian teratocarcinoma cancer cell line. Surprisingly, CH1/PA-1 cells showed a very low baseline level of NQO1 activity (Figure 10 and Figure S39). We assume that their lack of NQO1 activity might be a key to understanding their pronounced resistance to the tested compounds compared to SW480 cells.

## 4. Conclusions

Phthiocol (**L**) and complexes **1**–**6** with six different arenes, *p*-cymene (**1**), benzene (**2**), toluene (**3**), indane (**4**), biphenyl (**5**) and hexamethylbenzene (**6**), were successfully synthesized via microwave irradiation. This new set of tridentate phthiocol-based ruthenium arene complexes was characterized by using ^1^H-NMR and ^13^C-NMR spectroscopy, the crystal structures were examined by X-ray single-crystal spectroscopy. The aqueous stability of all complexes **1**–**6** was studied by HPLC-MS chromatography. DFT calculations were performed, and the theoretical results were in good agreement with the experimental stability data.

Cytotoxicity assays yielded remarkable results, as the broadly chemosensitive CH1/PA-1 teratocarcinoma cells were considerably less affected upon treatment with **L** and complexes **1**–**6** than SW480 colon carcinoma cells, which usually show moderate response to anticancer agents compared to CH1/PA-1 cells. The long-term cytotoxic effect was tested by a colony formation assay for 11 days, the results of which correspond well to those of the 24-h and 96-h MTT assays. Based on these data, the most promising compounds seem to be complexes **1** and **5** with a *p*-cymene and biphenyl arene, respectively. The cellular accumulation was determined by ICP-MS, where the most cytotoxic compounds, **1** and **5** (with the highest aqueous stability), yielded the highest total ruthenium content in the cell lysates. Moreover, complex **6** showed enhanced cellular accumulation with a similar magnitude as compounds **1** and **5**, despite the lowest stability in the aqueous milieu. A correlation could be found between cellular accumulation and lipophilicity, with complexes having higher calculated miLogP values for the arene showing significantly enhanced cellular ruthenium levels.

Since Pereyra et al. reviewed various aspects of the mechanisms of action of naphthoquinone derivatives, we focused on some of these, such as the influence on cell cycle, apoptosis induction, ROS generation, DNA damage and inhibition of NQO1 activity. As for cytotoxicity, the experimental evidence from cell cycle studies suggests that the compounds have a stronger impact on SW480 cells than on CH1/PA-1 cells. Data from the apoptosis assay revealed a pronounced increase of early and late apoptotic cells by complexes **1** and **5** in SW480 cells. Neither ROS generation nor a meaningful extent of interaction with DNA could be observed. Remarkably, NQO1 enzyme activity and its inhibition by some of the compounds at high concentrations could be detected only in SW480 but not CH1/PA-1 cells, which suggests that the unexpected resistance of CH1/PA-1 cells to the new agents might be due to a negligible baseline activity of this enzyme in the latter cell line.

## Figures and Tables

**Table 1:** Selected bond lengths of complexes **1**–**6** obtained X-ray single crystal diffraction [Å].

	Ru-*O*1	Ru-*O*2	Ru-*N*2	C1-*N*1	M-Arene
**1** ^(a)^	2.059(2)	2.121(2)	2.096(3)	1.528(4)	1.651
**2**	2.077(2)	2.086(2)	2.088(2)	1.514(3)	1.650
**3**	2.057(2)	2.104(2)	2.111(3)	1.507(4)	1.655
**4**	2.049(1)	2.116(1)	2.086(2)	1.516(2)	1.649
**6**	2.062(1)	2.117(1)	2.094(2)	1.523(2)	1.659

^(a)^ Bond lengths data from reference [17].

**Table 2:** Computed energies in [a.u.] of Ru-naphthoquinone-complexes in their initial- and transition-state, as well as their corresponding aqua complexes.

Arene	Reactant [a.u.]	Transition State [a.u.]	Aqua-Complex [a.u.]
*p*-Cymene (**1**)	−1356.045552	−1432.265348	−1432.285092
Benzene (**2**)	−1199.219445	−1275.437208	−1275.459690
Toluene (**3**)	−1238.431142	−1314.648960	−1314.670467
Indane (**4**)	−1315.656248	−1391.875753	−1391.894567
Biphenyl (**5**)	−1429.689526	−1505.909387	−1505.919352
HMB (**6**)	−1434.464410	−1510.686025	−1510.704607

**Table 3:** Cytotoxicity of compounds (**L**, **1**–**6**), 50% inhibitory concentrations in human carcinoma cell lines A549, SW480 and CH1/PA-1. Values are means ± SDs obtained by the MTT assay (exposure time: 24 h and 96 h), n.d. = not determined.

IC_50_ Values [µM]						
	A549		SW480		CH1/PA-1	
Compound	24 h	96 h	24 h	96 h	24 h	96 h
**1**	3.8 ± 2.6	1.2 ± 0.2 ^(a)^	0.46 ± 0.14	0.094 ± 0.031 ^(a)^	59 ± 18	>50 ^(a)^
**2**	>200	141 ± 38	31 ± 3	9.9 ± 2.5	170 ± 23	157 ± 8
**3**	>200	72 ± 12	11 ± 2	2.8 ± 0.7	121 ± 18	138 ± 10
**4**	119 ± 21	9.1 ± 1.5	3.5 ± 1.6	0.52 ± 0.09	146 ± 18	88 ± 10
**5**	4.3 ± 1.6	1.2 ± 0.2	0.77 ± 0.24	0.072 ± 0.019	42 ± 4	30 ± 3
**6**	38 ± 21	3.4 ± 0.6	2.8 ± 1.6	0.27 ± 0.06	57 ± 16	35 ± 4
**L**	>400	210 ± 32 ^(b)^	>400	116 ± 37 ^(b)^	>400	129 ± 29 ^(b)^
**[Ru(*p*-cym)Cl_2_]_2_^(c)^**	n.d.	>100	n.d.	>100	n.d.	>100
**[Ru(benzene)Cl_2_]_2_^(c)^**	n.d.	>100	n.d.	>100	n.d.	>100
**[Ru(toluene)Cl_2_]_2_^(c)^**	n.d.	>100	n.d.	>100	n.d.	>100
**[Ru(indane)Cl_2_]_2_^(c)^**	n.d.	257 ± 66	n.d.	166 ± 35	n.d.	51 ± 13
**Cisplatin**	n.d.	3.8 ± 1.0	n.d.	2.3 ± 0.2	n.d.	0.073 ± 0.001
**Carboplatin ^(d)^**	n.d.	38 ± 3	n.d.	42 ± 10	n.d.	0.79 ± 0.11
**Oxaliplatin ^(e)^**	n.d.	0.98 ± 0.21	n.d.	0.29 ± 0.05	n.d.	0.18 ± 0.01

^(a)^ IC_50_ values from reference [15]. ^(b)^ IC_50_ values from reference [17]. ^(c)^ IC_50_ values from reference [34]. ^(d)^ IC_50_ values from reference [35]. ^(e)^ IC_50_ values from reference [36].

## Data Availability

CCDC 2210525–2210528 contain the supplementary crystallographic data for this paper. These data can be obtained free of charge via www.ccdc.cam.ac.uk/data_request/cif, or by emailing data_request@ccdc.cam.ac.uk, or by contacting The Cambridge Crystallographic Data Centre, 12 Union Road, Cambridge CB2 1EZ, UK; fax: +44-1223-336033.

## Supplementary Materials

The following supporting information can be downloaded at: https://www.mdpi.com/article/10.3390/pharmaceutics14112466/s1. Materials, NMR spectra of complexes (Figures S1–S10), experimental parameters for X-ray analysis, crystal structures (Figures S11–S14), sample and crystal data, data collection, and structure refinement (Tables S1–S5), HPLC and HPLC-MS chromatograms (Figures S15–S20), calculated miLogP values of used arenes (Table S6), concentration–effect curves (Figures S21 and S22), correlation between IC_50_ values and miLogP values (Figure S23), colony formation assay (Table S7, Figures S24–S27), cellular accumulation results (Table S8, Figure S28); ICP-MS parameters (Table S9), cell cycle distribution and histograms (Tables S10 and S11, Figures S29–S32), apoptosis induction (Table S12, Figure S33), ROS assay results (Figures S34 and S35), dsDNA plasmid assay results (Figures S36 and S37) and NQO1 activity assay results (Figures S38 and S39). (References [49,50,51,52,53,54,55] are cited in the supplementary materials).
