# Supplementary material for: Arene Variation of Highly Cytotoxic Tridentate Naphthoquinone-Based Ruthenium(II) Complexes and In-Depth In Vitro Studies
Source: Pharmaceutics. 2022 Nov 15;14(11):2466. doi: 10.3390/pharmaceutics14112466 (PMC9699003; doi:10.3390/pharmaceutics14112466)
Supplement: Supplementary file 1 [file pharmaceutics-14-02466-s001.zip › pharmaceutics-1999801-supplementary.pdf]

Supporting information

Arene variation of highly cytotoxic tridentate naphthoquinone-based ruthenium(II) complexes and in-depth *in vitro* studies

Klaudia Cseh [1‡], Heiko Geisler [1‡], Kristina Stanojkovska [2], Julia Westermayr [3], Philipp Brunmayr [1], Dominik Wenisch [1], Natalie Gajic [1], Michaela Hejl [1], Martin Schaier [4,5], Gunda Koellensperger [4], Michael A. Jakupec [1,6], Philipp Marquetand [2,7] and Wolfgang Kandioller [1,6*]

[1] Institute of Inorganic Chemistry, Faculty of Chemistry, University of Vienna, Waehringer Str. 42, 1090 Vienna, Austria

[2] Institute of Theoretical Chemistry, Faculty of Chemistry, University of Vienna, Waehringer Str. 17, 1090 Vienna, Austria

[3] Wilhelm-Ostwald-Institute for Physical and Theoretical Chemistry, Faculty of Chemistry and Mineralogy, University of Leipzig, Linnéstr. 2, 04103 Leipzig, Germany

[4] Institute of Analytical Chemistry, Faculty of Chemistry, University of Vienna, Waehringer Str. 38, 1090 Vienna, Austria

[5] Vienna Doctoral School in Chemistry (DoSChem), University of Vienna, Waehringer Str. 42, 1090 Vienna, Austria

[6] Research Cluster "Translational Cancer Therapy Research", University of Vienna, 1090 Vienna, Austria

[7] Vienna Research Platform on Accelerating Photoreaction Discovery, University of Vienna, 1090 Vienna, Austria

‡ Contributed equally

* Correspondence: wolfgang.kandioller@univie.ac.at; Tel.: +43-1-4277-52609

Table of Contents

NMR spectra 1

X-Ray Diffraction Analysis 6

HPLC-MS data.. 11

Calculated miLogP values 13

Biological studies.. 14

MTT assay 14

Colony formation assay 17

Cellular accumulation 20

Cell cycle distribution.. 22

Apoptosis assay.. 28

ROS assay 30

dsDNA plasmid assay.. 32

NQO1 activity assay 34

References.. 36

NMR spectra

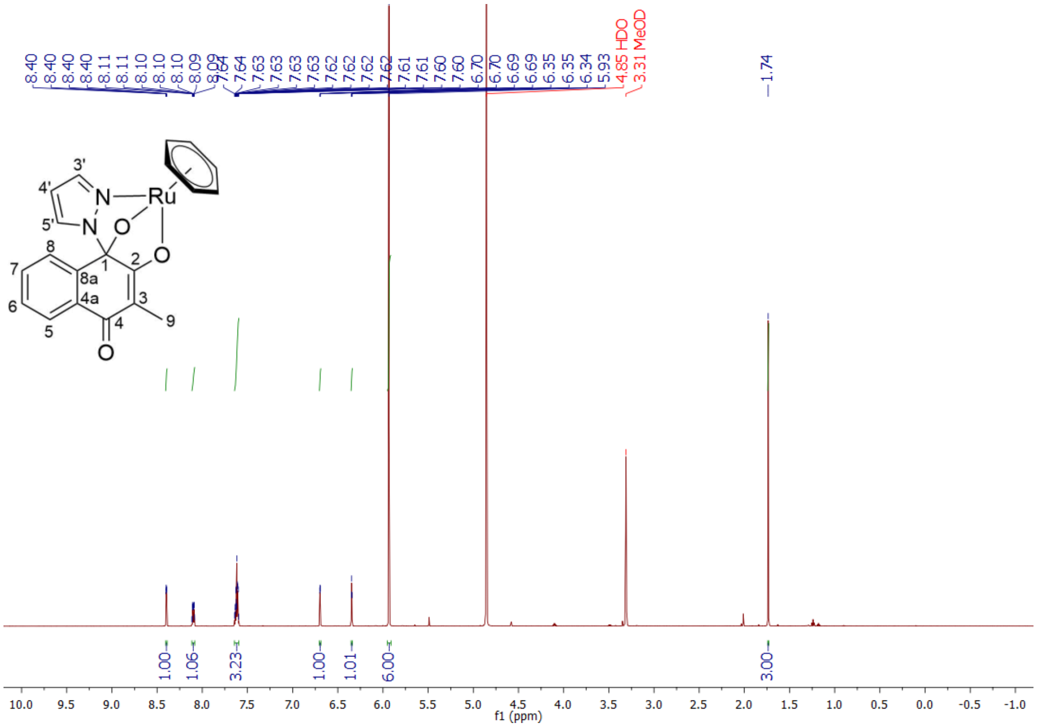

Figure S1: ^{1}H-NMR (MeOD-d_4, 600.25 MHz) spectrum of (**2**)

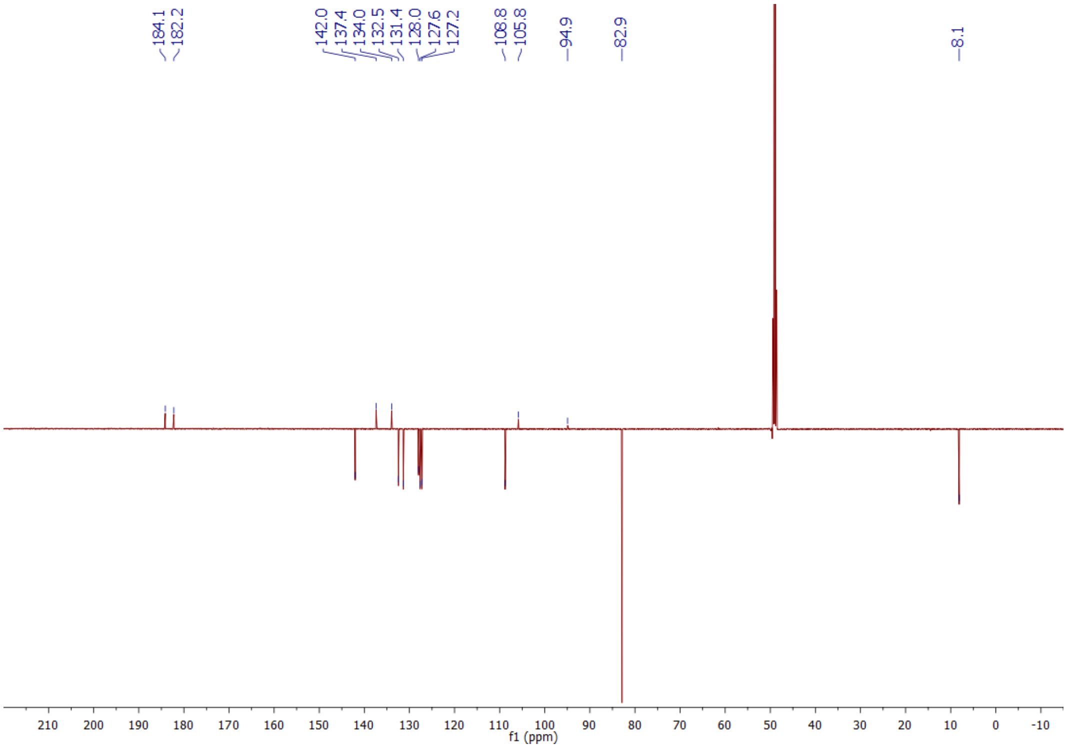

Figure S2: ^{13}C-NMR (MeOD-d_4, 150.93 MHz) spectrum of (**2**)

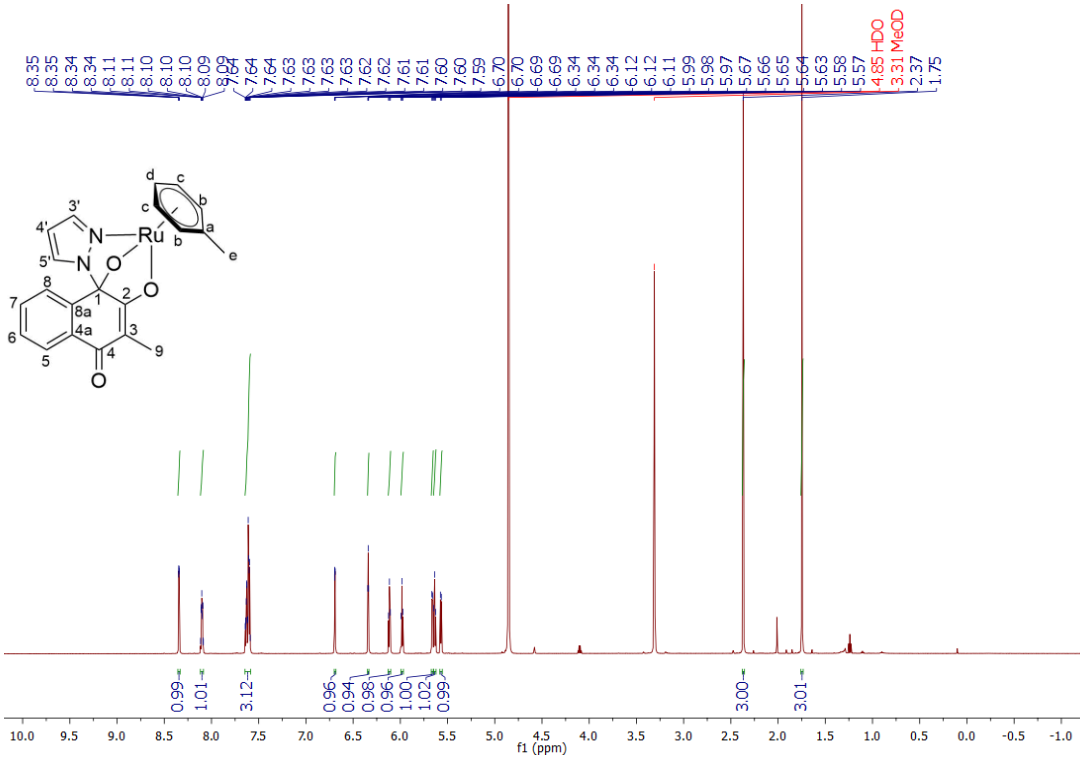

Figure S3: ^{1}H-NMR (MeOD-d_4, 600.25 MHz) spectrum of (**3**)

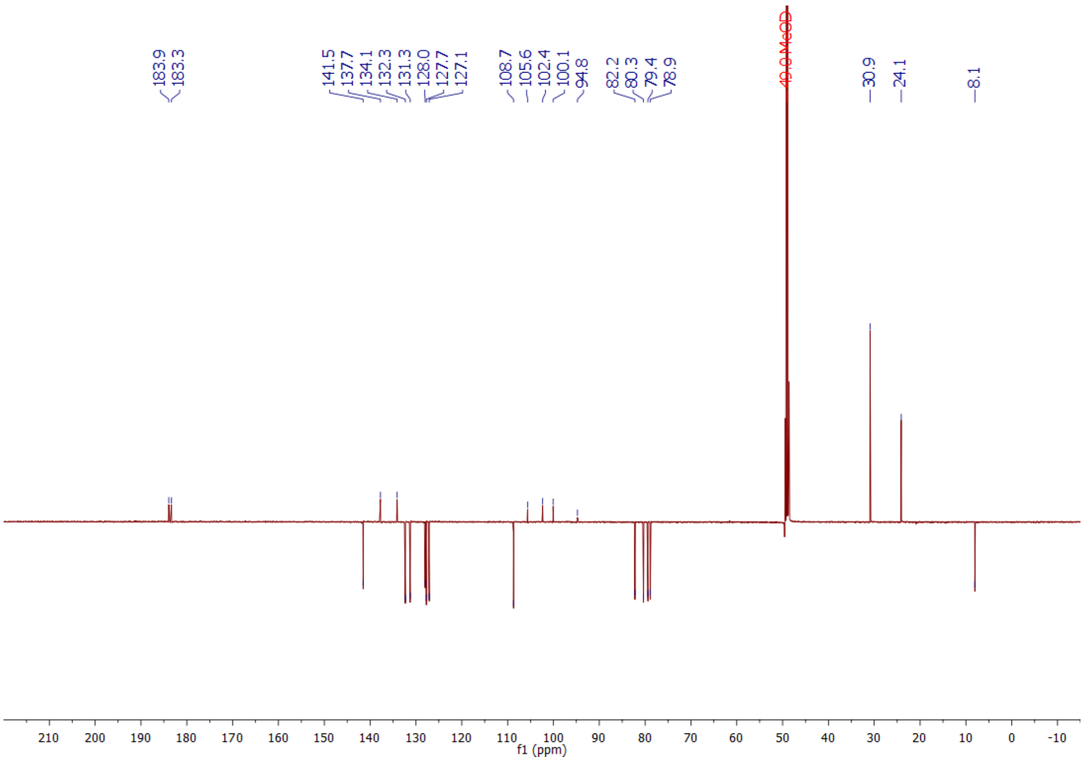

Figure S4: ^{13}C-NMR (MeOD-d_4, 150.93 MHz) spectrum of (**3**)

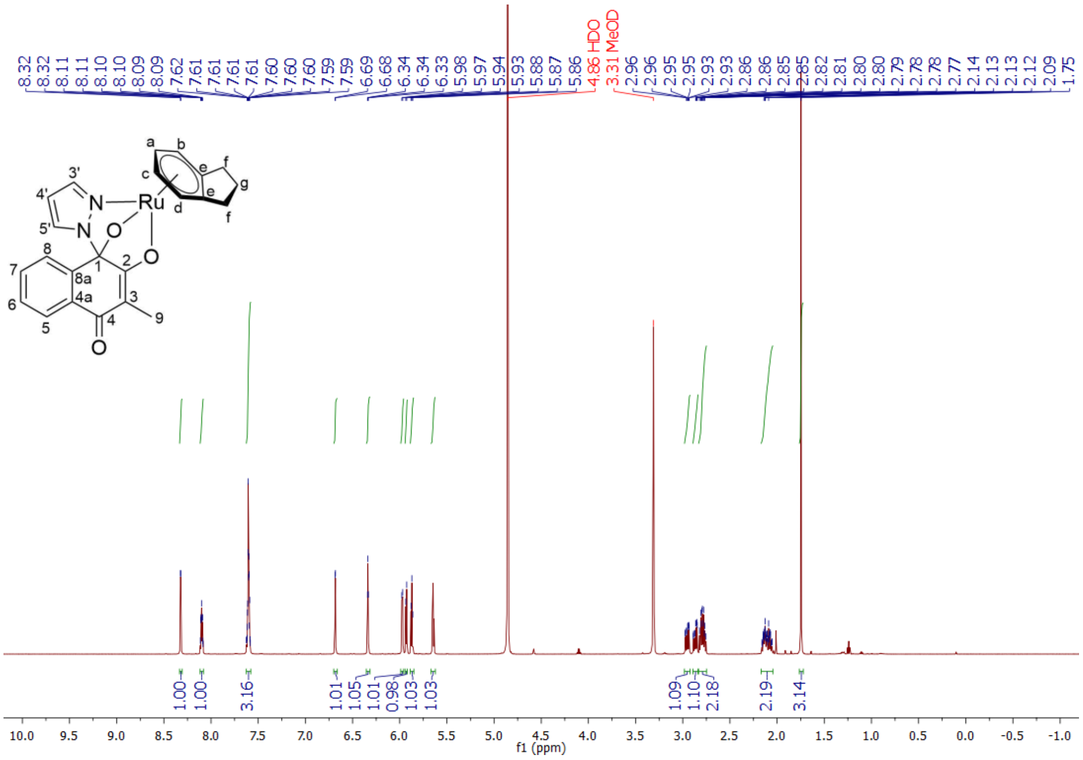

Figure S5: ^{1}H-NMR (MeOD-d_4, 600.25 MHz) spectrum of (**4**)

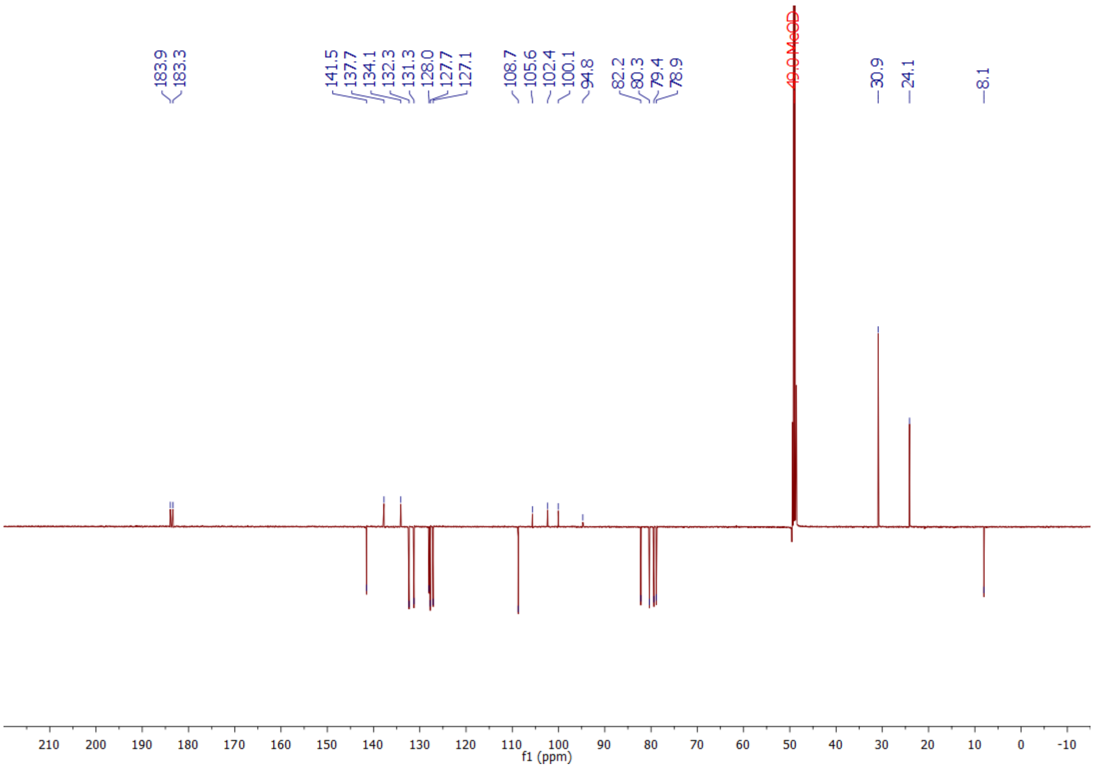

Figure S6: ^{13}C-NMR (MeOD-d_4, 150.93 MHz) spectrum of (**4**)

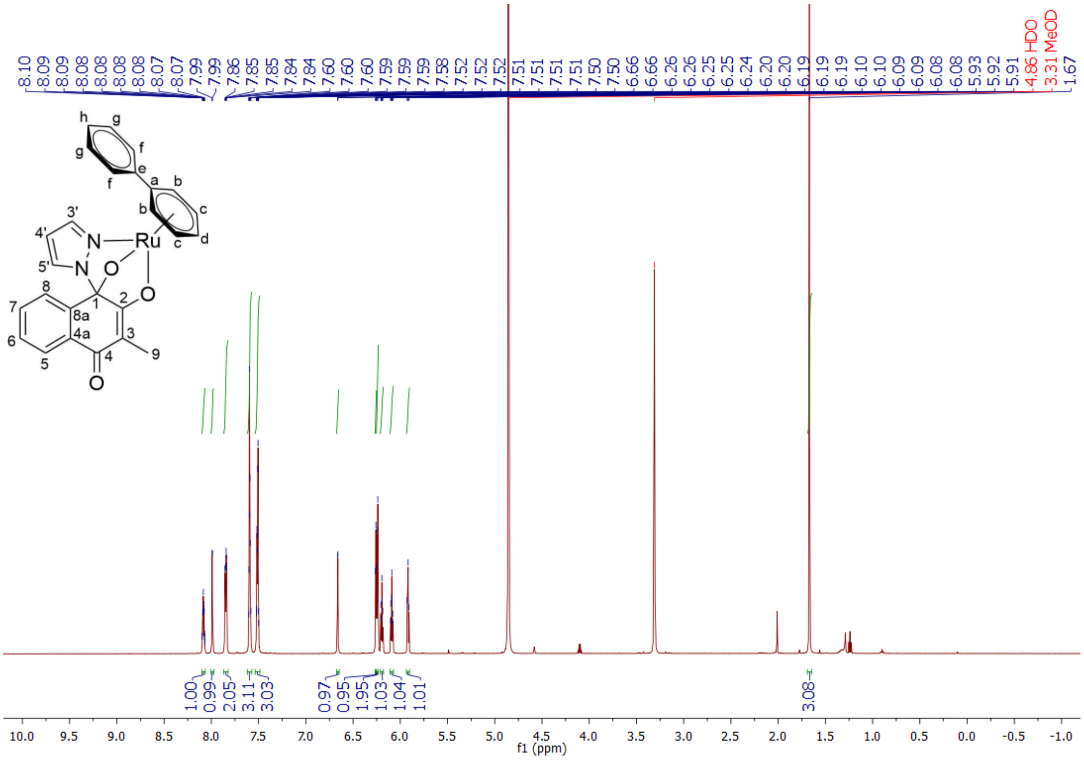

Figure S7: ^{1}H-NMR (MeOD-d_4, 600.25 MHz) spectrum of (**5**)

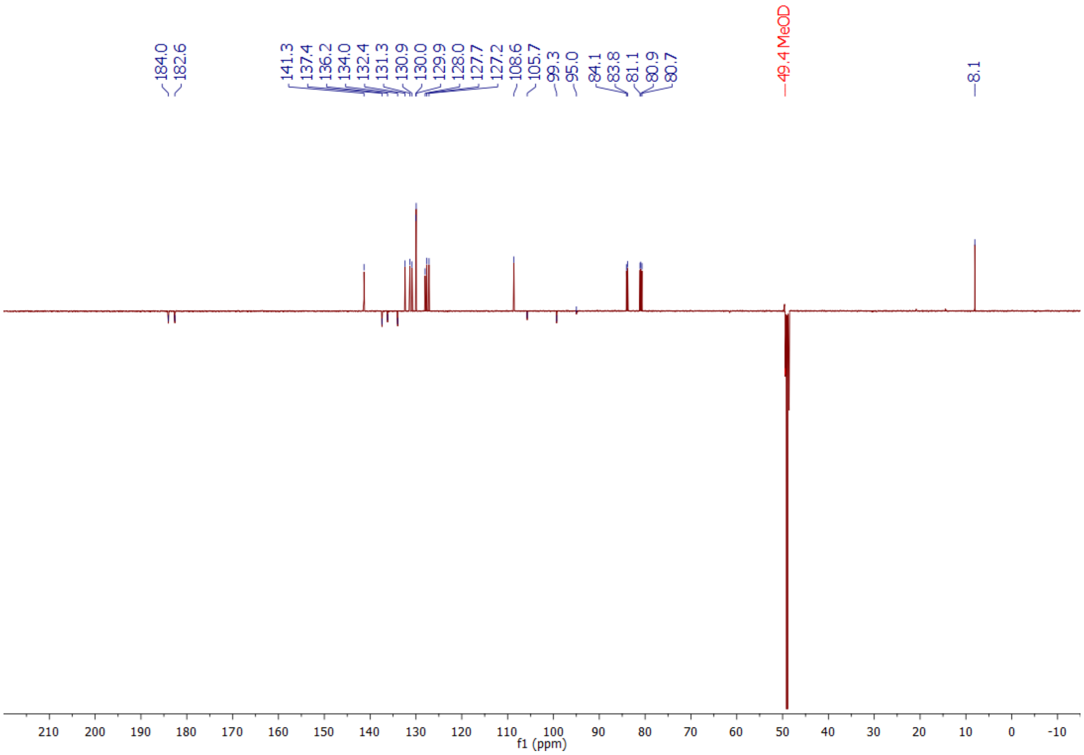

Figure S8: ^{13}C-NMR (MeOD-d_4, 150.93 MHz) spectrum of (**5**)

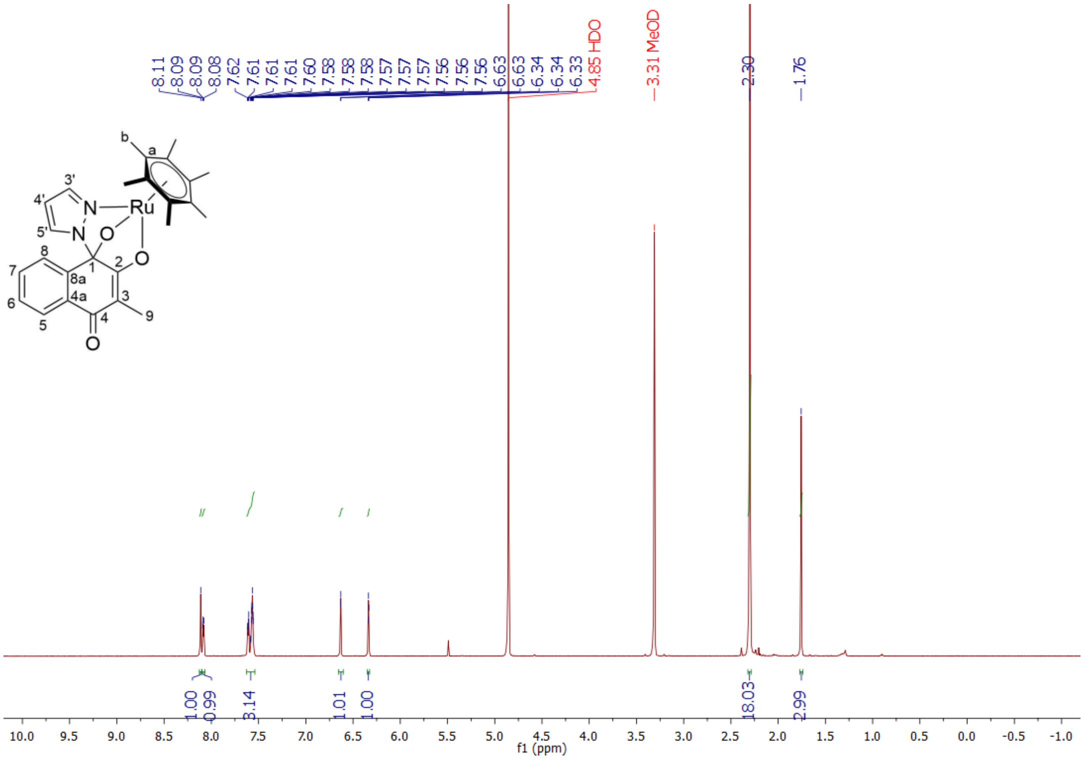

Figure S9: ^{1}H-NMR (MeOD-d_4, 700.40 MHz) spectrum of (**6**)

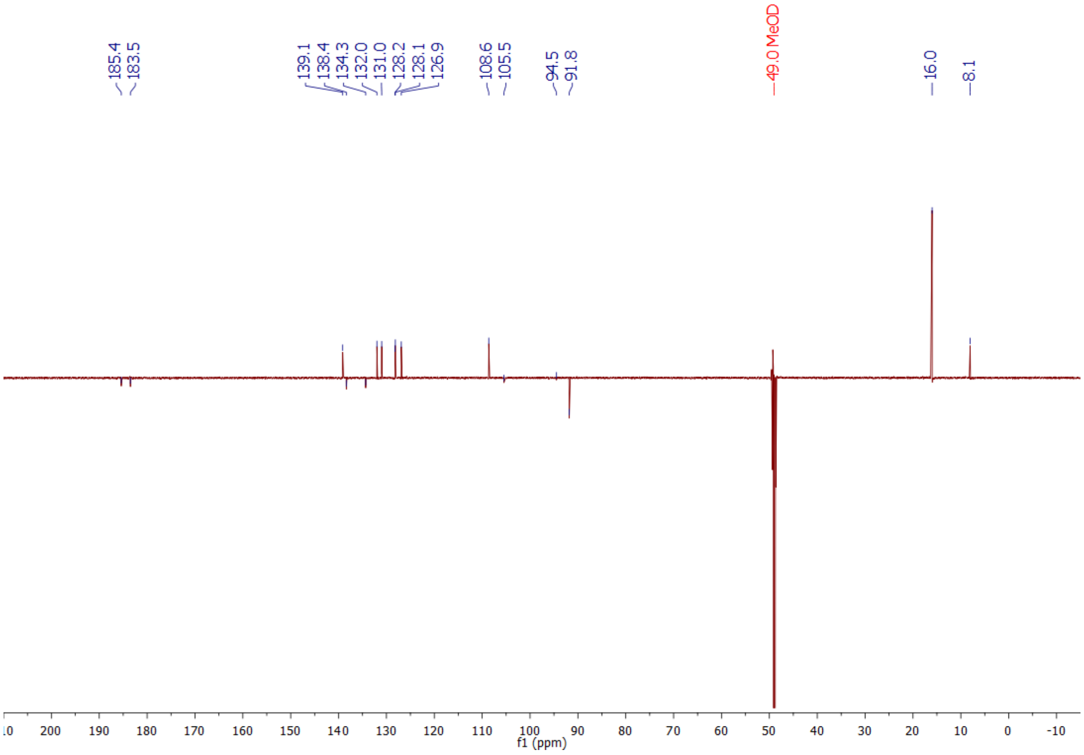

Figure S10: ^{13}C-NMR (MeOD-d_4, 176.13 MHz) spectrum of (**6**)

X-Ray Diffraction Analysis

The X-ray intensity data were measured on Bruker D8 Venture diffractometer equipped with multilayer monochromator, Mo K/α INCOATEC sealed tubes and Oxford cooling system. The structures were solved by *Direct and Iterative Methods*. Non-hydrogen atoms were refined with *anisotropic displacement parameters*. Hydrogen atoms were inserted at calculated positions and refined with riding model. The following software was used: *Bruker SAINT software package* [1] using a narrow-frame algorithm for frame integration, *SADABS* [2] for absorption correction, *OLEX2* [3] for structure solution, refinement, molecular diagrams and graphical user-interface, *Shelxle* [4] for refinement and graphical user-interface, *SHELXS-2015* [5] for structure solution, *SHELXL-2015* [6] for refinement, *Platon* [7] for symmetry check. Experimental data and CCDC-Codes Experimental data (Available online: http://www.ccdc.cam.ac.uk/conts/retrieving.html) can be found in Table S1. Crystal data, data collection parameters, and structure refinement details are given in Tables S2–S5. Asymmetric Units visualized in Figure S11–S14.

Table S1: Experimental parameter and CCDC-Code.

**Sample**	**Machine**	**Source**	**Temp.**	**Detector Distance**	**Time/ Frame**	**#Frames**	**Frame width**	**CCDC**
			[K]	[mm]	[s]		[°]	
**2**	Bruker D8	Mo	100	40	10	360	0.5	2210525
**3**	Bruker D8	Mo	100	40	15	209	0.5	2210526
**4**	Bruker D8	Mo	100	40	10	2403	0.5	2210527
**6**	Bruker D8	Mo	100	36	1	1896	0.36	2210528

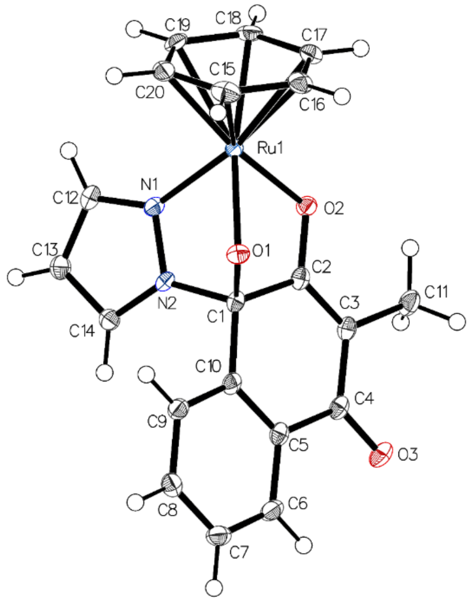

Figure S11: Asymmetric Unit drawn with 50% displacement ellipsoid. Solvent omitted for clarity. The bond precision for C-C single bonds is 0.0037 Å.

Table S2: Sample and crystal data, data collection and structure refinement

**Identification code**	mo_hege_PhBr004_P21n	**b [Å]**	9.7557(8)
**Empirical formula**	$C_{20}H_{18}N_2O_3Ru$	**c [Å]**	14.7947(11)
**Formula weight [g/mol]**	451.43	**α [°]**	90
**Temperature [K]**	100.0	**β [°]**	94.244(5)
**Crystal system**	monoclinic	**γ [°]**	90
**Space group**	P21/n	**Volume[Å^3]**	1691.2(3)
**a [Å]**	11.7496(12)	**Z**	4
**ρ_{calc}. [g/cm^3]**	1.773	**Reflections collected**	9577
**μ [mm^{-1}]**	0.958	**Independent reflections**	3074 [Rint = 0.0377, Rsigma = 0.0406]
**F(000)**	912.0	**Data/restraints/parameters**	3074/0/248
**Crystal size[mm^3]**	0.1 × 0.09 × 0.04	**Goodness-of-fit on F^2**	1.066
**Radiation**	MoKα (λ = 0.71073)	**Final R indexes [I>=2σ (I)]**	R1 = 0.0246, wR2 = 0.0619
**2Θ range for data collection [°]**	4.596 to 50.686	**Final R indexes [all data]**	R1 = 0.0274, wR2 = 0.0640
**Index ranges**	-14 ≤ h ≤ 14, -11 ≤ k ≤ 11, -17 ≤ l ≤ 17	**Largest diff. peak/hole [e$^-$ Å^{-3}]**	0.49/-0.42

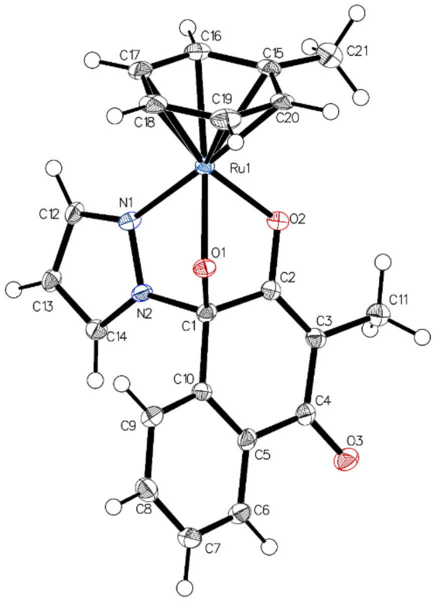

Figure S12: Asymmetric Unit drawn with 50% displacement ellipsoid. Solvent omitted for clarity. The bond precision for C-C single bonds is 0.0046 Å.

Table S3: Sample and crystal data, data collection and structure refinement

**Identification code**	mo_hege_PhBr005_Ibca	**b [Å]**	18.5085(11)
**Empirical formula**	$C_{21}H_{19}N_2O_3Ru$	**c [Å]**	24.2359(14)
**Formula weight [g/mol]**	456.45	**α [°]**	90
**Temperature [K]**	100.0	**β [°]**	90
**Crystal system**	orthorhombic	**γ [°]**	90
**Space group**	Ibca	**Volume[Å$^{3]}$**	7146.1(7)
**a [Å]**	15.9307(8)	**Z**	16
**ρ_{calc}. [g/cm^3]**	1.697	**Reflections collected**	12602
**μ [mm^{-1}]**	0.906	**Independent reflections**	3276 [Rint = 0.0638, Rsigma = 0.0508]
**F(000)**	3696.0	**Data/restraints/parameters**	3276/0/254
**Crystal size[mm^3]**	0.06 × 0.04 × 0.02	**Goodness-of-fit on F^2**	1.019
**Radiation**	MoKα (λ = 0.71073)	**Final R indexes [I>=2σ (I)]**	R1 = 0.0330, wR2 = 0.0706
**2Θ range for data collection [°]**	4.762 to 50.692	**Final R indexes [all data]**	R1 = 0.0451, wR2 = 0.0783
**Index ranges**	-19 ≤ h ≤ 17, -21 ≤ k ≤ 22, -26 ≤ l ≤ 29	**Largest diff. peak/hole [e$^-$ Å^{-3}]**	0.38/-0.56

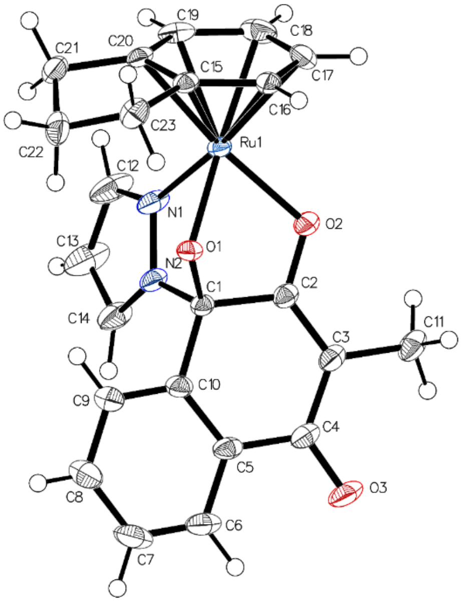

Figure S13: Asymmetric Unit drawn with 50% displacement ellipsoid. The bond precision for C-C single bonds is 0.0027 Å.

Table S4: Sample and crystal data, data collection and structure refinement

**Identification code**	mo_hege_PhBr015_P-1	**b [Å]**	9.9658(4)
**Empirical formula**	$C_{23}H_{20}N_2O_3Ru$	**c [Å]**	14.1016(5)
**Formula weight [g/mol]**	473.48	**α [°]**	96.1229(14)
**Temperature [K]**	100.0	**β [°]**	104.6352(14)
**Crystal system**	triclinic	**γ [°]**	101.1567(14)
**Space group**	P-1	**Volume[Å^{3}]**	1047.14(7)
**a [Å]**	7.9569(3)	**Z**	2
**$\rho_{calc.}$ [g/cm^3]**	1.502	**Reflections collected**	51436
**μ [mm^{-1}]**	0.774	**Independent reflections**	6115 [R_{int} = 0.0371, R_{sigma} = 0.0208]
**F(000)**	480.0	**Data/restraints/parameters**	6115/0/263
**Crystal size[mm^3]**	0.09 × 0.07 × 0.05	**Goodness-of-fit on F^2**	1.053
**Radiation**	MoKα (λ = 0.71073)	**Final R indexes [I>=2σ (I)]**	R_1 = 0.0248, wR_2 = 0.0579
**2Θ range for data collection [°]**	4.776 to 60.114	**Final R indexes [all data]**	R_1 = 0.0278, wR_2 = 0.0596
**Index ranges**	-11 ≤ h ≤ 11, -14 ≤ k ≤ 14, -19 ≤ l ≤ 19	**Largest diff. peak/hole [e$^-$ Å^{-3}]**	0.87/-0.66

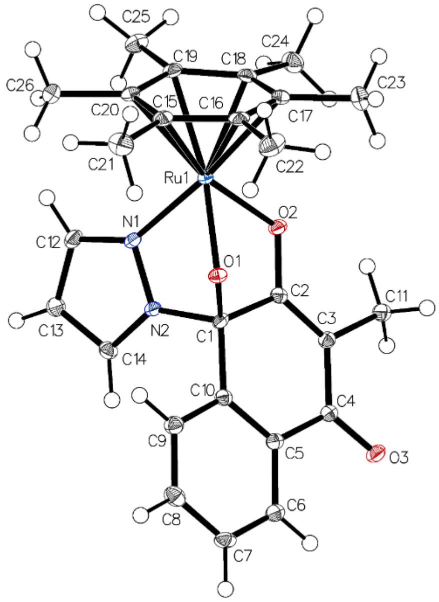

Figure S14: Asymmetric Unit drawn with 50% displacement ellipsoid. Solvent omitted for clarity. The bond precision for C-C single bonds is 0.0023 Å. A solvent mask was calculated, and 40 electrons were found in a volume of 130 Å^3 in 1 void per unit cell. This is consistent with the presence of 2[H_2O] per Unit Cell which account for 40 electrons per unit cell.

Table S5: Sample and crystal data, data collection and structure refinement

**Identification code**	HeGe764_P21n	**b [Å]**	20.8855(3)
**Empirical formula**	$C_{27}H_{30}Cl_2N_2O_3Ru$	**c [Å]**	14.4325(2)
**Formula weight [g/mol]**	602.50	**α [°]**	90
**Temperature [K]**	100.0	**β [°]**	105.1468(6)
**Crystal system**	monoclinic	**γ [°]**	90
**Space group**	$P2_1/n$	**Volume[Å^3]**	2470.99(6)
**a [Å]**	8.49260(10)	**Z**	4
**$\rho_{calc.}$ [g/cm^3]**	1.620	**Reflections collected**	73651
**μ [mm^{-1}]**	0.884	**Independent reflections**	7494 [R_{int} = 0.0659, R_{sigma} = 0.0383]
**F(000)**	1232.0	**Data/restraints/parameters**	7494/6/342
**Crystal size[mm^3]**	0.176 × 0.16 × 0.087	**Goodness-of-fit on F^2**	1.035
**Radiation**	MoKα (λ = 0.71073)	**Final R indexes [I>=2σ (I)]**	R_1 = 0.0287, wR_2 = 0.0680
**2Θ range for data collection [°]**	4.874 to 61.09	**Final R indexes [all data]**	R_1 = 0.0451, wR_2 = 0.0726
**Index ranges**	-12 ≤ h ≤ 12, -29 ≤ k ≤ 29, -17 ≤ l ≤ 20	**Largest diff. peak/hole [e$^-$ Å^{-3}]**	0.64/-0.78

HPLC-MS data

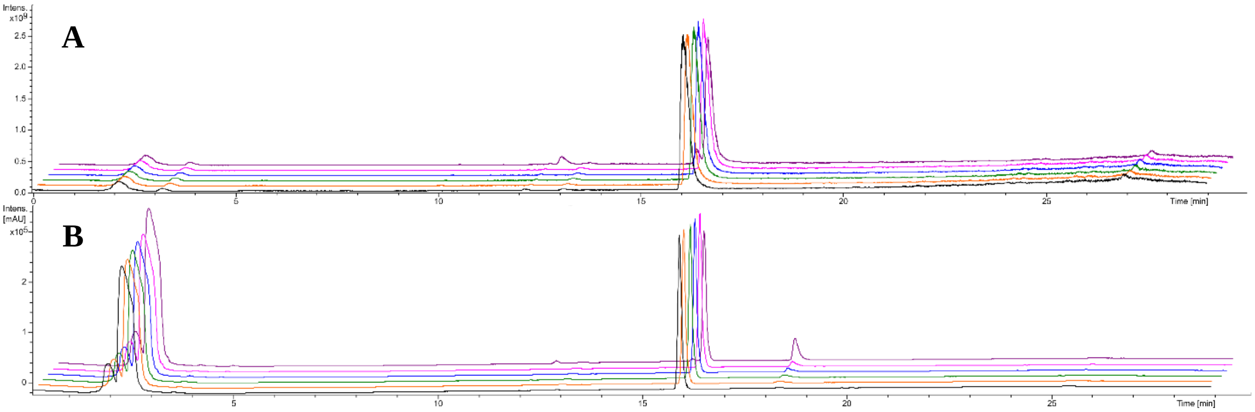

Figure S15: HPLC-MS chromatogram (A) and HPLC chromatogram (B) of compound 1 after 0, 1, 2, 3, 4, 24 h (from front to back; conditions see experimental section).

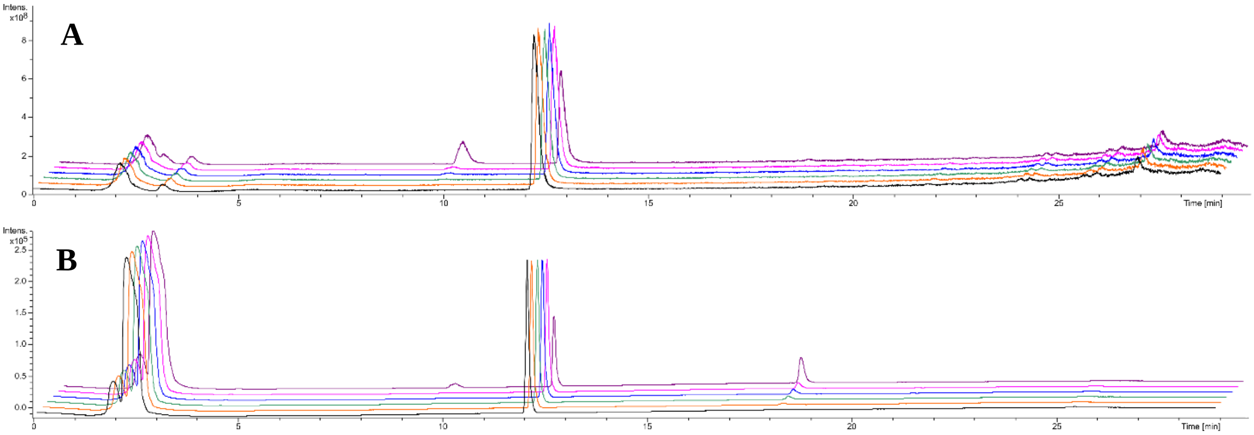

Figure S16: HPLC-MS chromatogram (**A**) and HPLC chromatogram (**B**) of compound **2** after 0, 1, 2, 3, 4, 24 h (from front to back; conditions see experimental section).

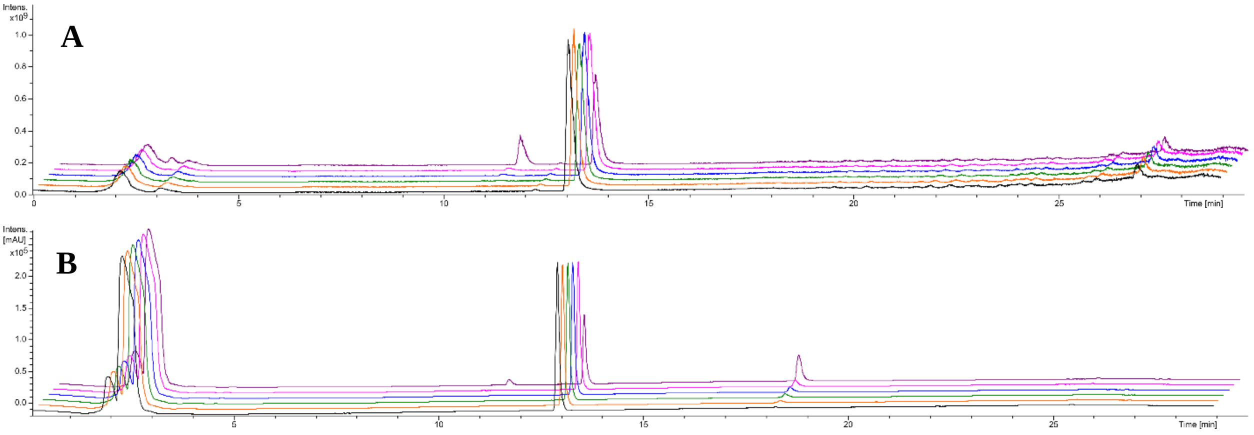

Figure S17: HPLC-MS chromatogram (**A**) and HPLC chromatogram (**B**) of compound **3** after 0, 1, 2, 3, 4, 24 h (from front to back; conditions see experimental section).

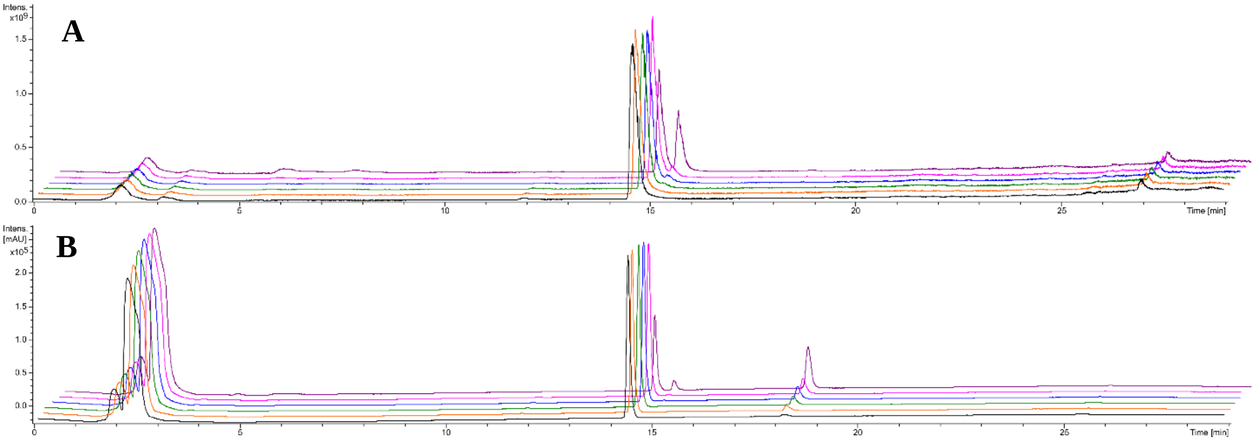

Figure S18: HPLC chromatogram-MS (**A**) and HPLC chromatogram (**B**) of compound **4** after 0, 1, 2, 3, 4, 24 h (from front to back; conditions see experimental section).

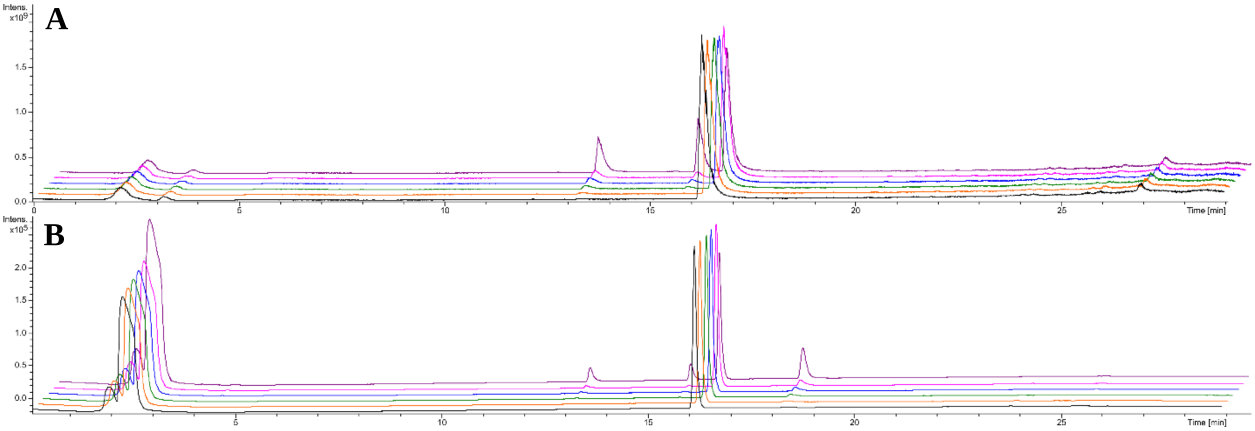

Figure S19: HPLC chromatogram-MS (**A**) and HPLC chromatogram (**B**) of compound **5** after 0, 1, 2, 3, 4, 24 h (from front to back; conditions see experimental section).

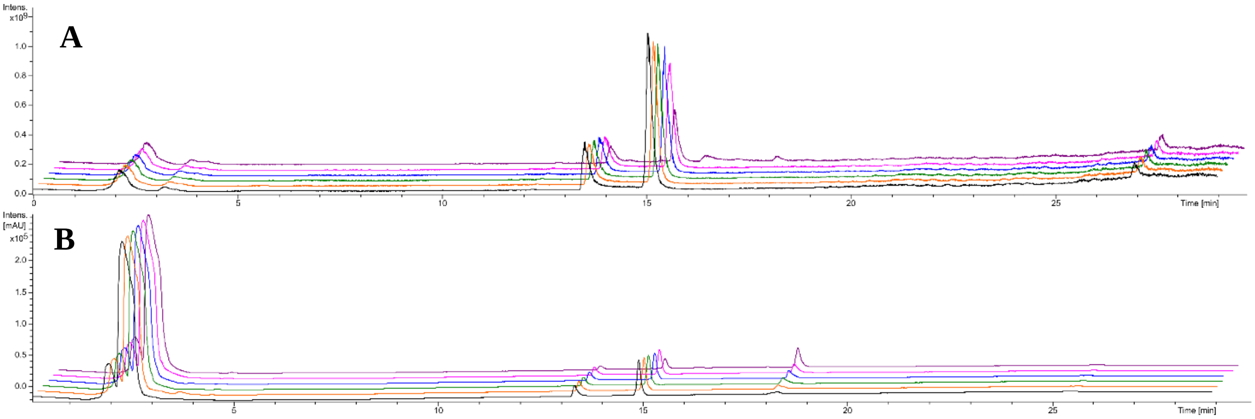

Figure S20: HPLC chromatogram-MS (**A**) and HPLC chromatogram (**B**) of compound **6** after 0, 1, 2, 3, 4, 24 h (from front to back; conditions see experimental section).

Calculated miLogP values

Table S6: Calculated miLogP values of used arenes. Calculations were performed at www.molinspiration.com

Arene	miLogP	Arene	miLogP
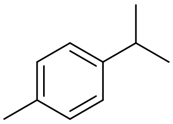 *p*-Cymene	3.90	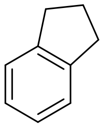 Indane	2.38
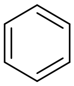 Benzene	1.94	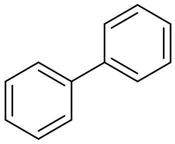 Biphenyl	3.73
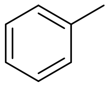 Toluene	2.39	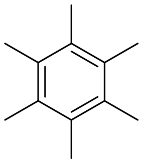 Hexamethylbenzene (HMB)	4.34

Biological studies

MTT assay

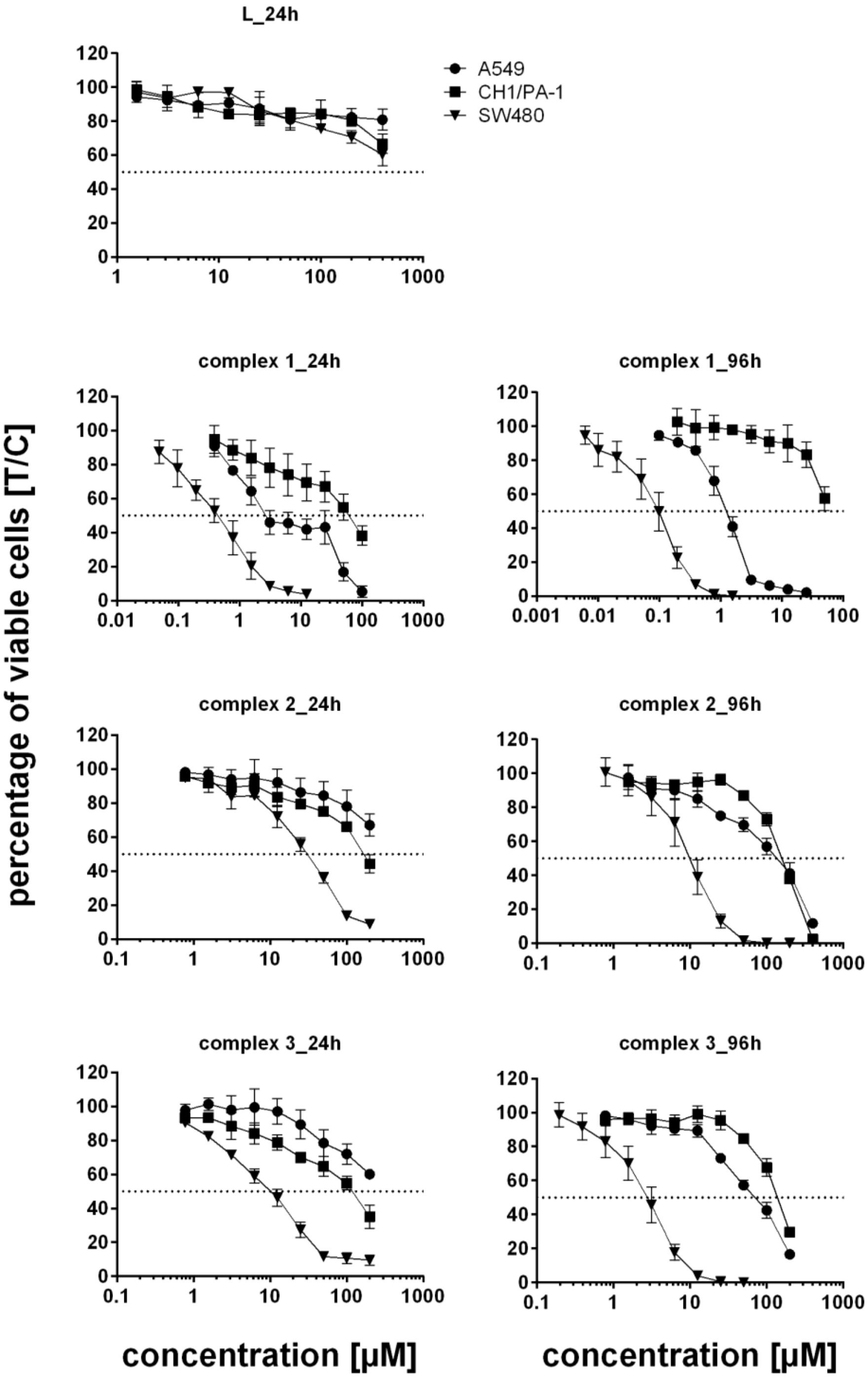

Figure S21: Concentration-effect curves of compounds **L** and **1–3** in three cell lines A549, SW480 and CH1/PA-1 after 24 h and 96 h exposure in the MTT assay.

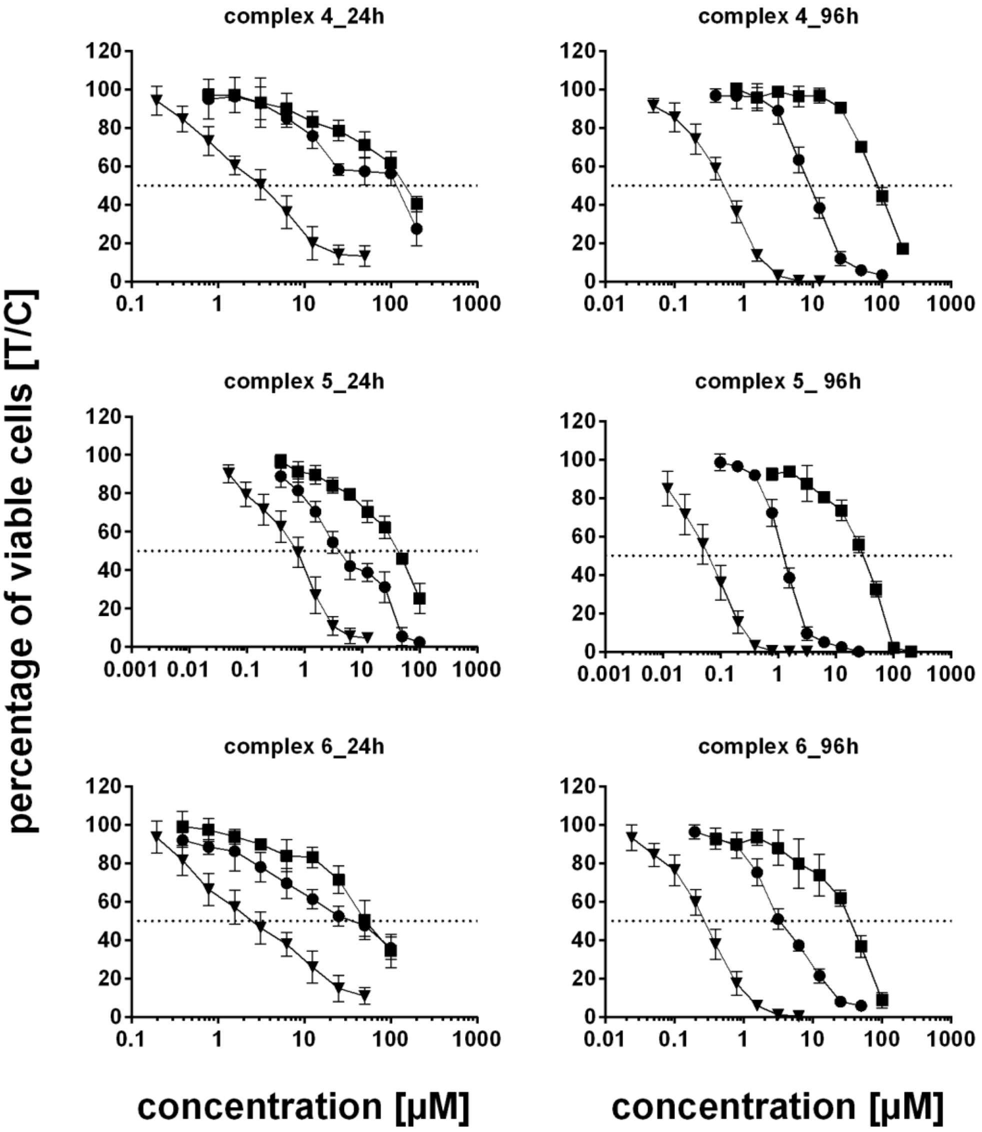

Figure S22: Concentration-effect curves of compounds **4–6** in three cell lines A549, SW480 and CH1/PA-1 after 24 h and 96 h exposure in the MTT assay.

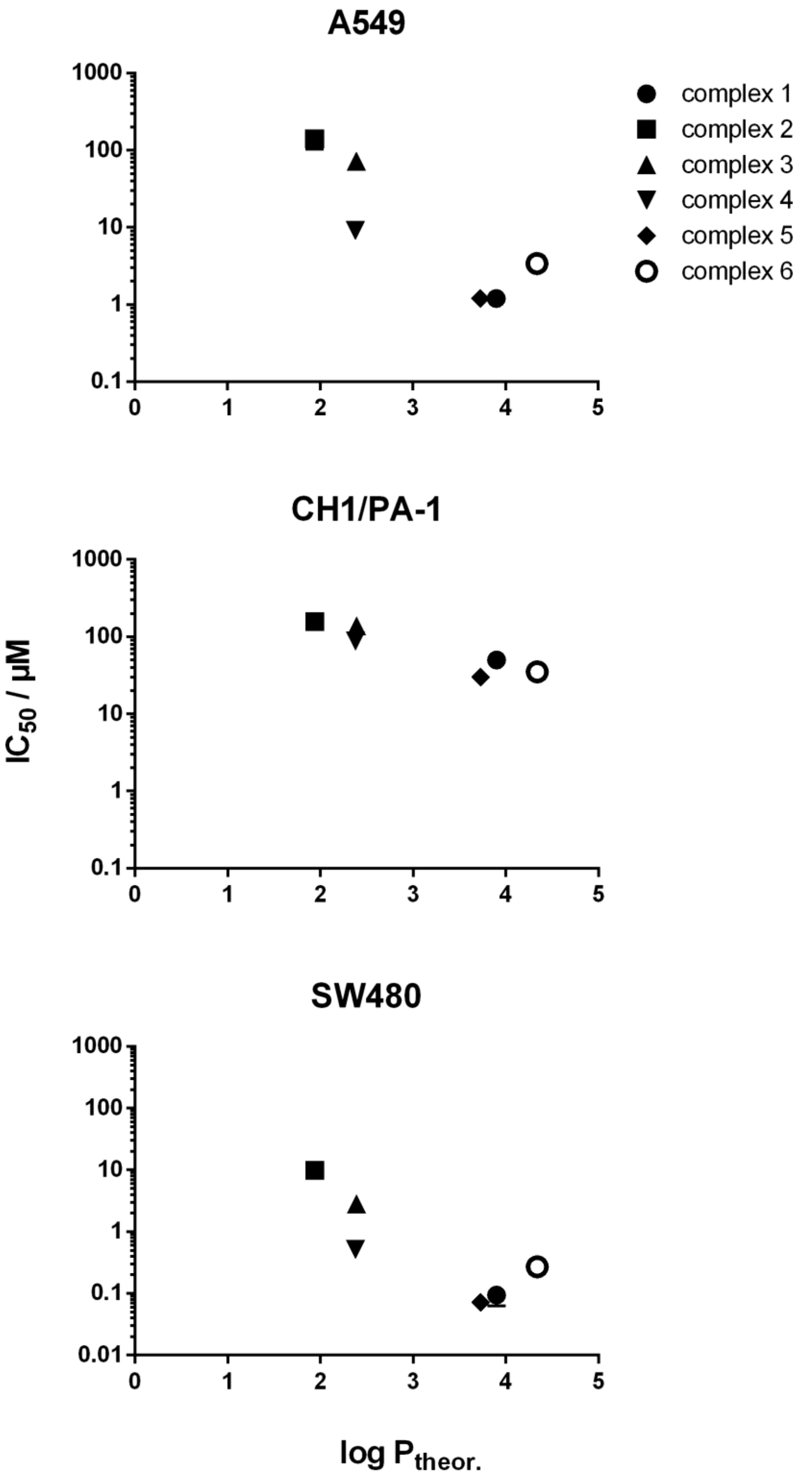

Figure S23: Correlation between IC_{50} values (96 h exposure) and theoretical lipophilicity coefficient in A549, CH1/PA-1 and SW480 cells.

Colony formation assay

Table S7: Impact of compounds **1–6** and **L** on colony formation in SW480 cells. Data are means ± standard deviations of three independent experiments and normalized to the negative control. (In negative controls 168 ± 18 colonies were counted.)

Compound	Concentration [μM]	Number of colonies [%]
control	0	100
1	0.039	18 ± 5
	0.078	3.1 ± 0.8
	0.16	0.26 ± 0.43
2	5	48 ± 9
	10	20 ± 4
	20	3.5 ± 1.5
3	1.25	53 ± 8
	2.5	23 ± 8
	5	4.3 ± 3.7
4	0.31	22 ± 5
	0.63	5.1 ± 2.1
	1.25	0.82 ± 0.85
5	0.039	19 ± 7
	0.078	4.0 ± 2.0
	0.16	0.22 ± 0.48
6	0.16	28 ± 12
	0.31	9.2 ± 3.2
	0.63	1.2 ± 1.1
L	50	62 ± 25
	100	0 ± 0
	150	0 ± 0

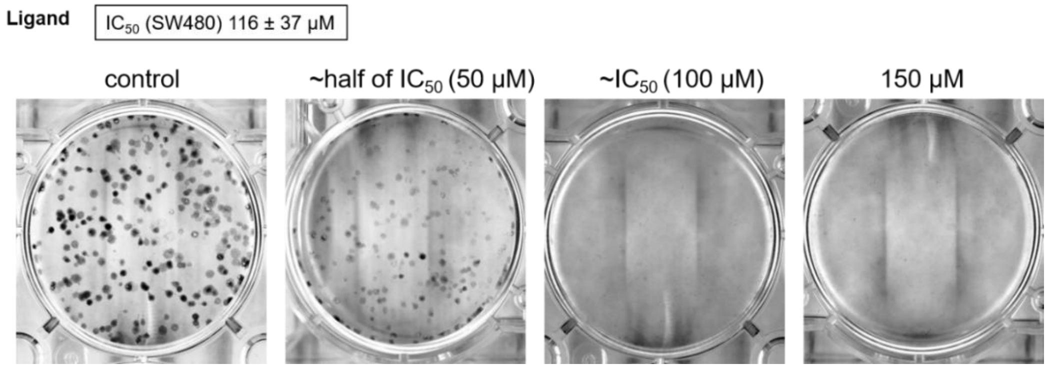

Figure S24: Representative images of the colony formation assay with compound **L**, which reduced the long-term proliferation capacity of SW480 cells *in vitro*. Chosen concentrations are based on 96-h IC_{50} values in the MTT assay.

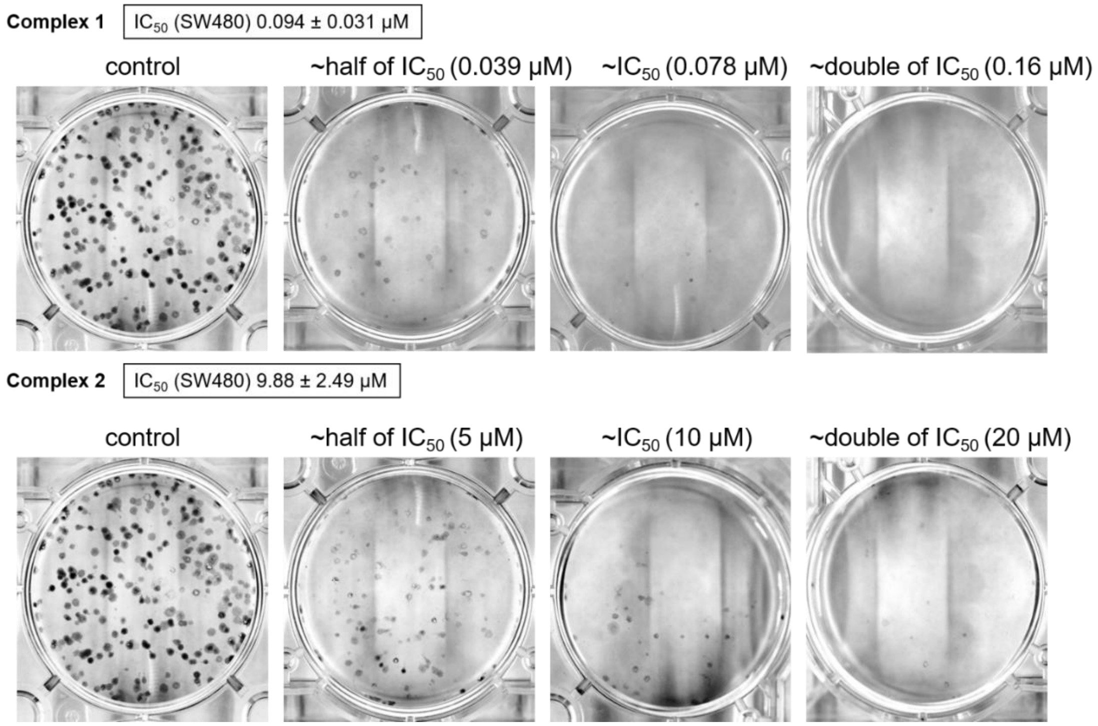

Figure S25: Representative images of the colony formation assay with compounds **1** and **2**, which reduced the long-term proliferation capacity of SW480 cells *in vitro*. Chosen concentrations are based on 96-h IC_{50} values in the MTT assay.

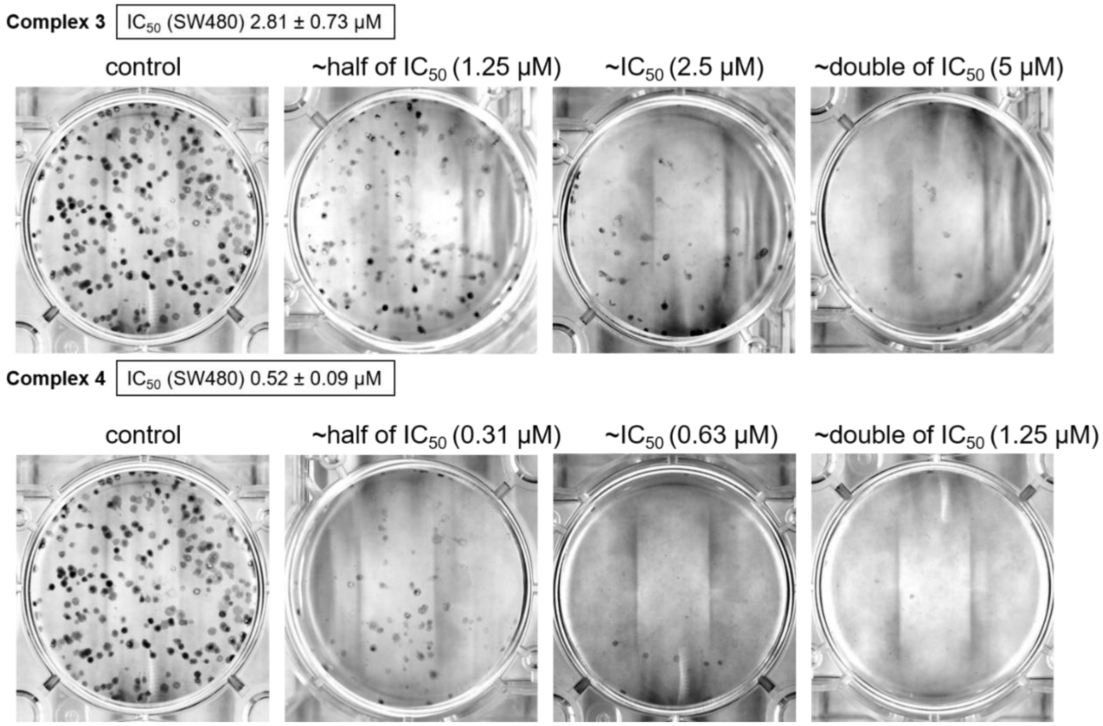

Figure S26: Representative images of the colony formation assay with compounds **3** and **4**, which reduced the long-term proliferation capacity of SW480 cells *in vitro*. Chosen concentrations are based on 96-h IC_{50} values in the MTT assay.

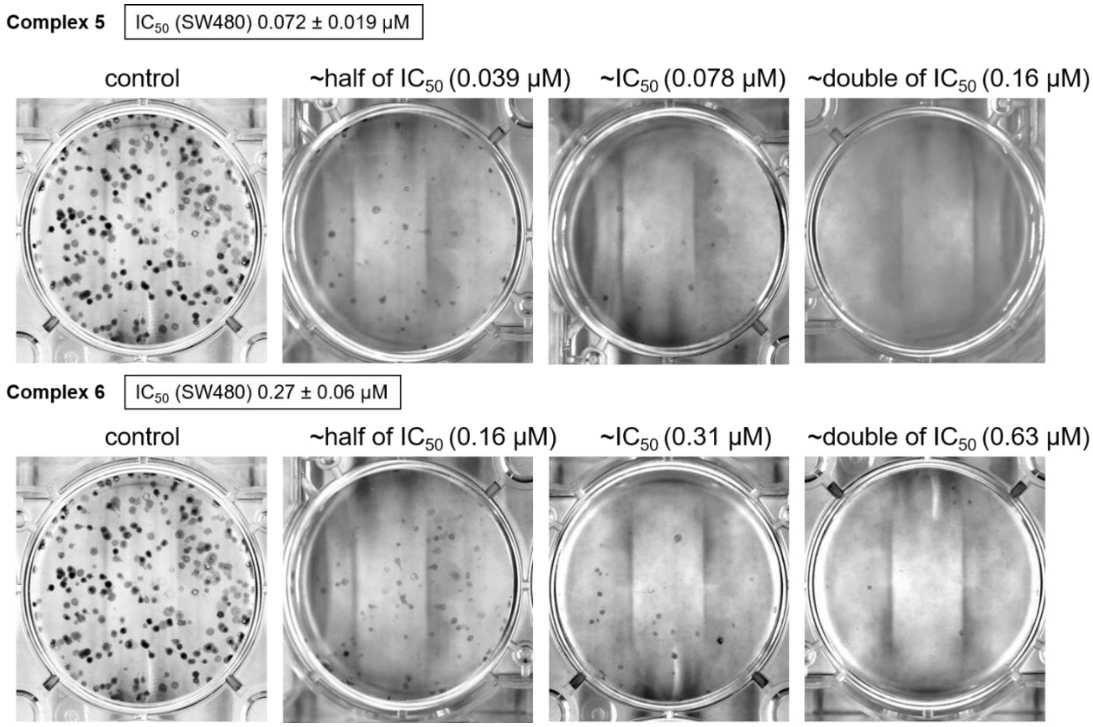

Figure S27: Representative images of the colony formation assay with compounds **5** and **6**, which reduced the long-term proliferation capacity of SW480 cells *in vitro*. Chosen concentrations are based on 96-h IC_{50} values in the MTT assay.

Cellular accumulation

Table S8: Cellular accumulation in SW480 cells after 2 h of incubation with the tested complexes **1–6** at 37 °C and two different concentrations (2 µM and 5 µM).

Compound	Concentration [µM]	Ru [102]/cell [fg]
**1**	2	97 ± 8
	5	299 ± 54
**2**	2	4.5 ± 0.3
	5	11 ± 2
**3**	2	15 ± 2
	5	33 ± 10
**4**	2	44 ± 3
	5	100 ± 15
**5**	2	107 ± 14
	5	336 ± 10
**6**	2	100 ± 15
	5	206 ± 32

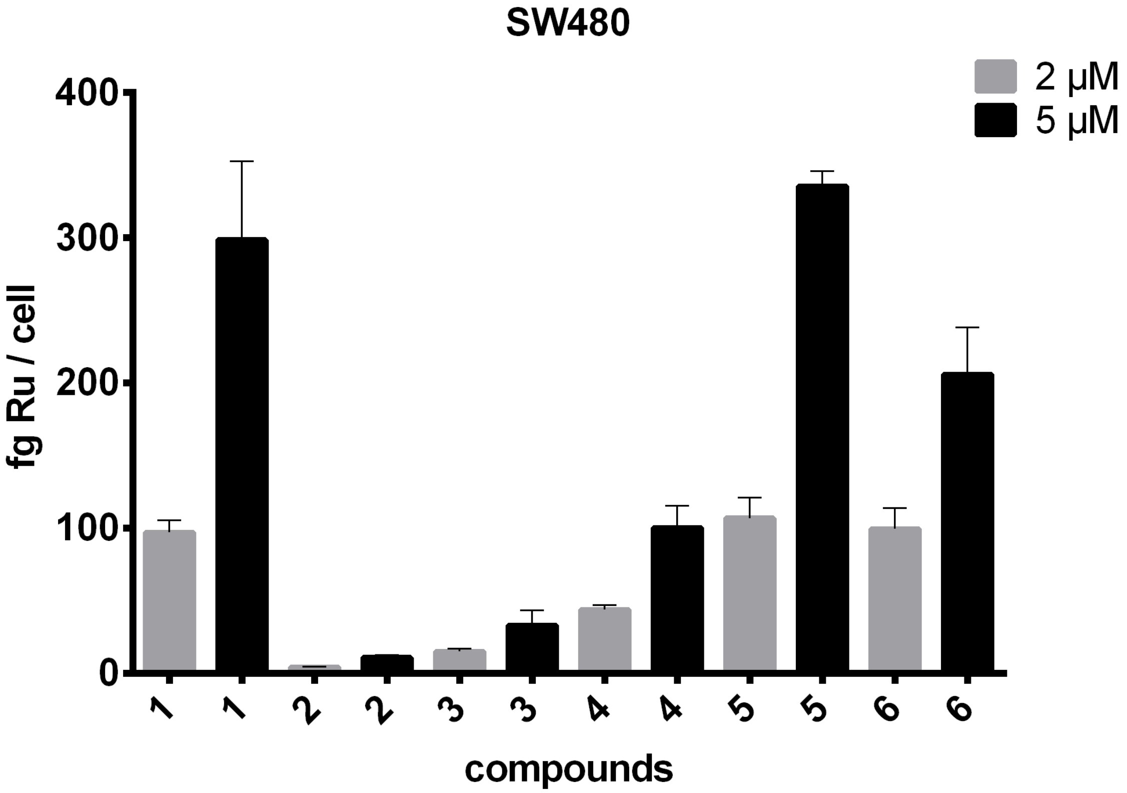

Figure S28: Cellular ruthenium accumulation of compounds **1–6** in fg Ru/cell, measured by ICP-MS. SW480 cells were treated for 2 h with 2 µM and 5 µM of test solutions. Values are means ± SDs from at least three independent measurements performed in triplicates.

Table S9: Instrumental parameters for the determination of the ruthenium content using ICP-MS.

RF power [W]	1550
Nebulizer	MicroMist
Spray chamber	Scott double-pass
Spray chamber temp. [°C]	2
Cone materials	Ni
Plasma gas flow (Ar) [L min^{-1}]	15.0
Auxiliary gas flow (Ar) [L min^{-1}]	0.90
Nebulizer gas flow (Ar) [L min^{-1}]	1.03
Monitored Isotopes	^{102}Ru, ^{115}In
Integration time [s]	0.1

Cell cycle distribution

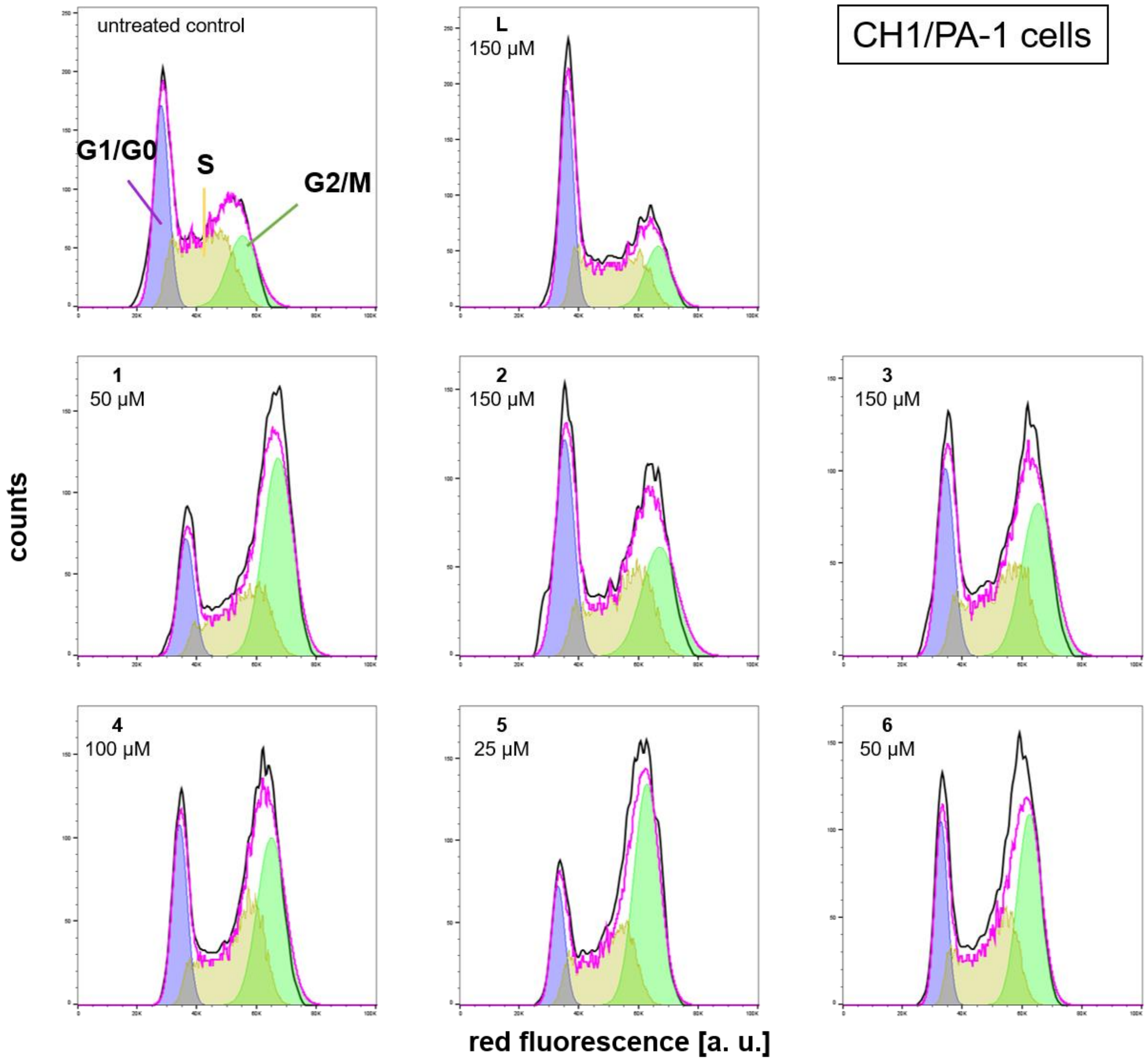

Figure S29: Histograms of cell cycle phase distribution in CH1/PA-1 cells after 24 h drug exposure. The cells were exposed to **L** and complexes **1–6** in a subcytotoxic concentration (around IC_{50} values from 96 h MTT assays). The top left histogram represents an untreated control. All histograms are from one representative experiment out of at least three repetitions per tested concentration.

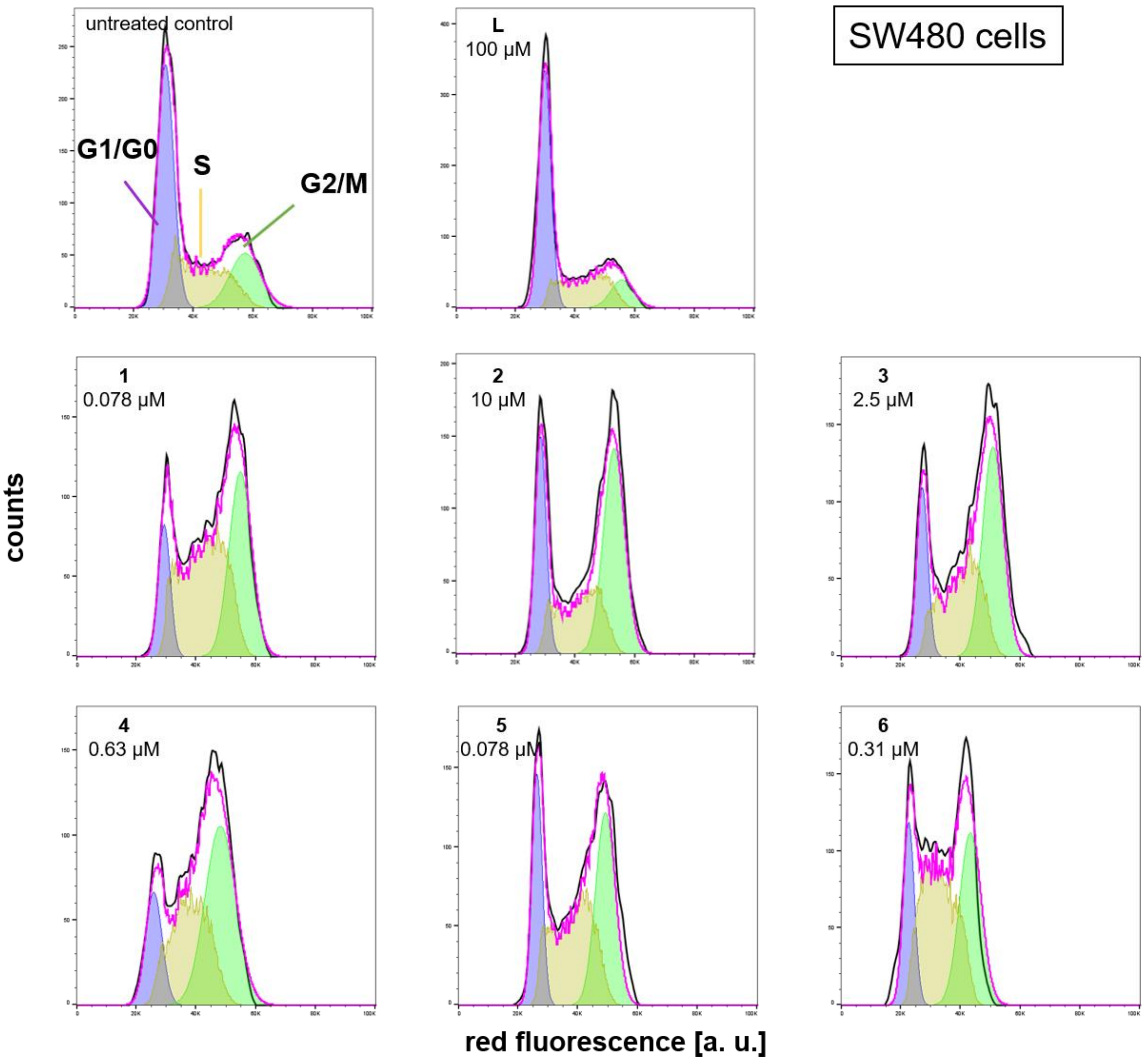

Figure S30: Histograms of cell cycle phase distribution in SW480 cells after 24 h drug exposure. The cells were exposed to **L** and complexes **1–6** in a subcytotoxic concentration (around IC_{50} values from 96 h MTT assays). The top left histogram represents an untreated control. All histograms are from one representative experiment out of at least three repetitions per tested concentration.

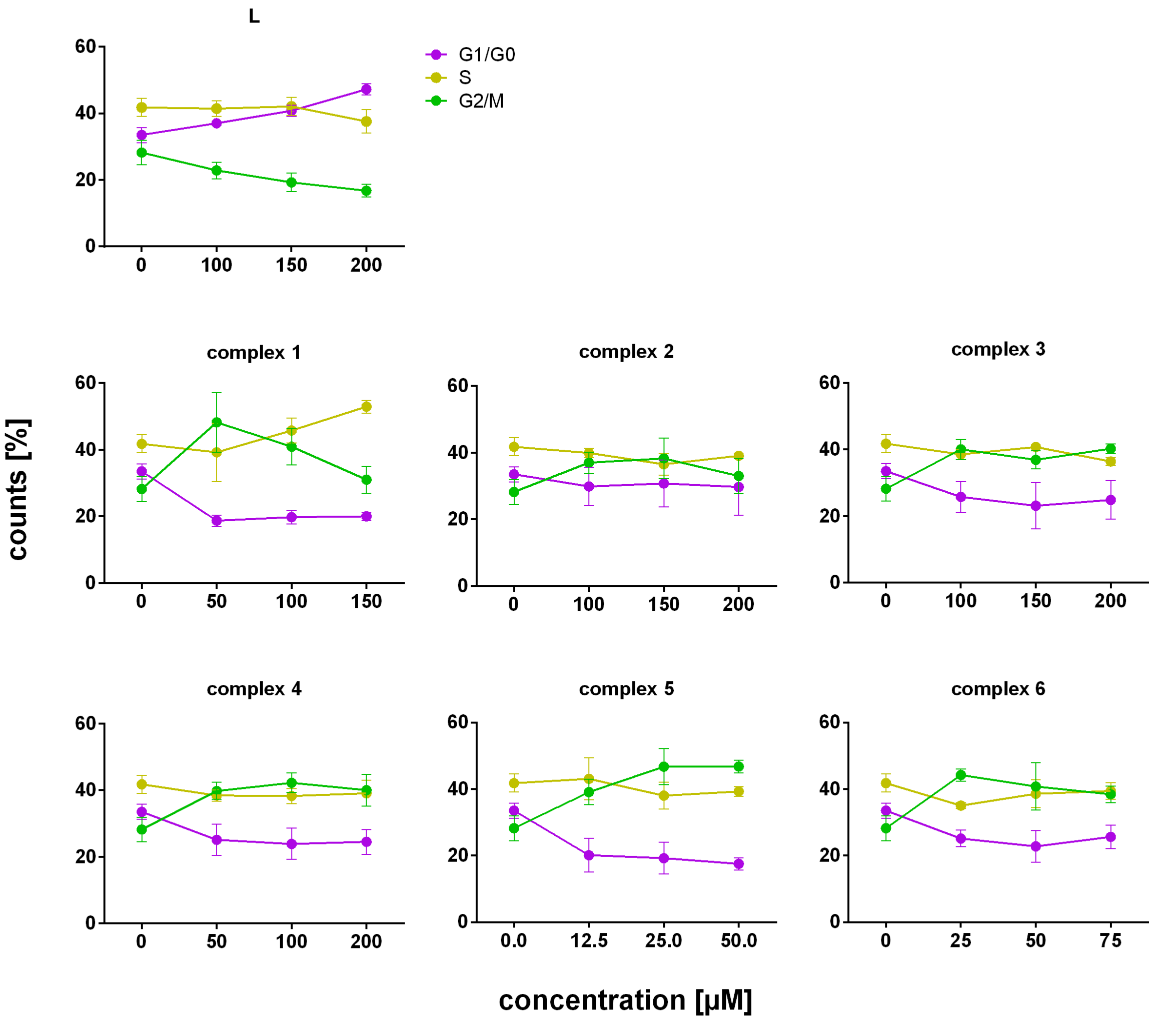

Figure S31: Impact of **L** and complexes **1–6** on cell cycle distribution in CH1/PA-1 cells. Graphs indicate the percentages (means ± SDs) of cells in G1/G0, S and G2/M phases of the cell cycle.

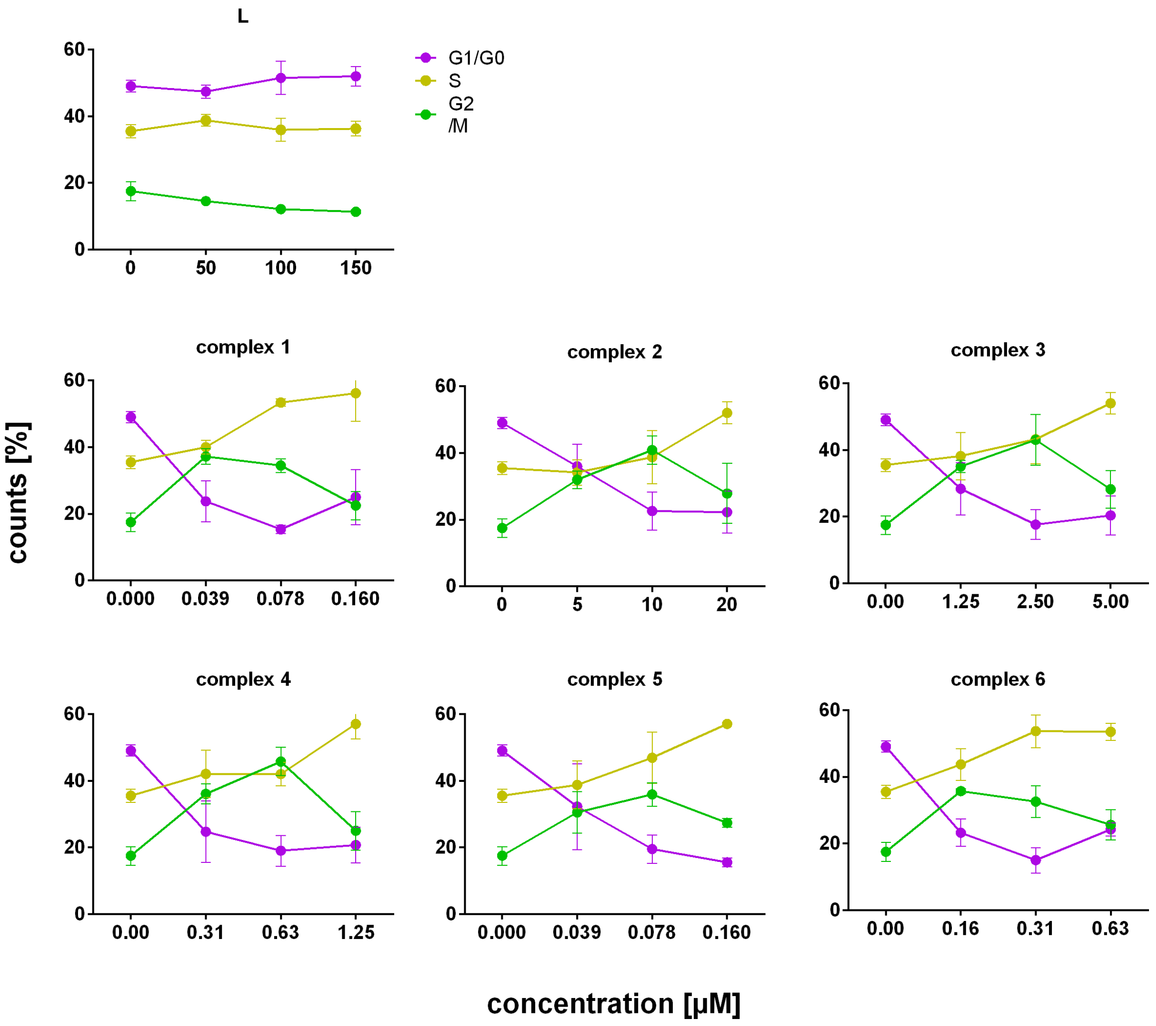

Figure S32: Impact of **L** and complexes **1–6** on cell cycle distribution in SW480 cells. Graphs indicate the percentages (means ± SDs) of cells in G1/G0, S and G2/M phases of the cell cycle.

Table S10: Cell cycle alterations in CH1/PA-1 cells treated with compounds **L** and **1–6** at three different concentrations (around the IC_{50} values based on 96 h MTT assays). Data are mean percentages ± SDs of at least three experiments.

**CH1/PA-1**		Cell cycle phase		
Compound	Concentration [µM]	G1/G0	S	G2/M
Control	0	33.5 ± 2.3	41.8 ± 2.7	28.2 ± 2.5
**L**	100	37.0 ± 0.3	41.4 ± 2.4	22.9 ± 2.8
	150	40.8 ±1.7	42.1 ± 2.7	19.3 ± 1.9
	200	47.2 ± 1.7	37.6 ± 3.5	16.8 ± 8.9
**1**	50	18.7 ± 1.7	39.2 ± 8.7	48.2 ± 5.5
	100	19.8 ± 2.1	45.8 ± 3.8	40.9 ± 4.0
	150	20.0 ± 1.3	52.9 ± 1.9	31.0 ± 3.2
**2**	100	29.9 ± 5.7	39.9 ± 1.4	37.0 ± 6.1
	150	30.8 ± 7.1	36.5 ± 3.3	38.3 ± 5.3
	200	29.7 ± 8.5	39.1 ± 0.8	33.0 ± 3.0
**3**	100	25.8 ± 4.6	38.6 ± 1.5	40.1 ± 2.7
	150	23.1 ±7.0	40.8 ± 0.6	36.9 ± 1.5
	200	24.9 ± 5.8	36.4 ± 1.2	40.3 ± 2.6
**4**	50	25.1 ± 4.7	38.4 ± 1.7	39.8 ± 2.9
	100	23.9 ± 4.7	38.3 ± 2.3	42.2 ± 4.7
	200	24.5 ± 3.8	39.1 ± 3.9	40.0 ± 3.8
**5**	12.5	20.1 ± 5.0	43.1 ± 6.3	39.1 ± 5.4
	25	19.2 ± 4.8	38.0 ± 4.0	46.7 ± 1.9
	50	17.5 ± 1.8	39.3 ± 1.4	46.8 ± 1.8
**6**	25	25.1 ± 2.5	35.0 ± 0.9	44.2 ± 7.1
	50	22.8 ± 4.7	38.6 ± 4.2	40.8 ± 2.5
	75	25.6 ± 3.5	39.4 ± 2.5	38.4 ± 3.7

Table S11: Cell cycle alterations in SW480 cells treated with compounds **L** and **1–6** at three different concentrations (around the IC_{50} values based on 96 h MTT assays). Data are mean percentages ± SDs of at least three experiments.

**SW480**		Cell cycle phase		
Compound	Concentration [µM]	G1/G0	S	G2/M
Control	0	49.1 ± 1.7	35.5 ± 1.9	17.5 ± 2.8
**L**	50	47.4 ± 2.0	38.8 ± 1.7	14.5 ± 0.5
	100	51.5 ± 5.0	35.9 ± 3.4	12.1 ± 0.4
	150	52.0 ± 2.9	36.3 ± 2.2	11.3 ± 0.5
**1**	0.039	23.8 ± 6.1	40.0 ± 2.1	37.2 ± 2.3
	0.078	15.4 ± 1.2	53.4 ± 1.1	34.5 ± 2.1
	0.16	25.0 ± 8.2	56.2 ± 8.4	22.5 ± 4.3
**2**	5	36.0 ± 6.7	34.2 ± 3.8	32.0 ± 2.6
	10	22.6 ± 5.7	38.8 ± 7.9	40.9 ± 4.2
	20	22.3 ± 6.3	52.1 ± 3.3	27.9 ± 9.0
**3**	1.25	28.4 ± 7.8	38.2 ± 7.1	35.1 ± 1.8
	2.5	17.6 ± 4.5	43.3 ± 7.4	43.1 ± 7.6
	5	20.4 ± 5.9	54.1 ± 3.2	28.2 ± 5.6
**4**	0.31	24.7 ± 9.2	42.1 ± 7.1	36.1 ± 3.0
	0.63	19.0 ± 4.6	42.0 ± 3.5	45.8 ± 4.2
	1.25	20.7 ± 5.3	57.1 ± 4.5	25.0 ± 5.8
**5**	0.039	32.3 ± 12.9	38.8 ± 7.2	30.5 ± 6.2
	0.078	19.5 ± 4.3	46.9 ± 7.7	35.9 ± 3.5
	0.16	15.5 ± 1.3	57.1 ± 0.1	27.4 ± 1.3
**6**	0.16	23.2 ± 4.1	43.7 ± 4.8	35.7 ± 0.7
	0.31	15.0 ± 3.8	53.7 ± 4.9	32.6 ± 4.7
	0.63	24.2 ± 2.0	53.5 ± 2.6	25.6 ± 4.5

Apoptosis assay

Table S12: Apoptosis and necrosis induction, examined by annexin V-FITC/PI staining. SW480 colon cancer cells were exposed to compounds **L** and **1–6** at the indicated concentrations for 24 h and cell populations were analyzed by flow cytometry. Results are mean percentages ± standard deviations of at least three independent experiments.

Compound	Concentration (µM)		mean ± SD
Control	0	necrotic cells [%]	2.0 ± 1.6
		late apoptotic cells [%]	3.5 ±1.5
		early apoptotic cells [%]	2.9 ± 2.3
		viable cells [%]	92 ± 4
**1**	0.1	necrotic cells [%]	0.77 ± 0.33
		late apoptotic cells [%]	3.6 ± 2.6
		early apoptotic cells [%]	3.7 ± 3.1
		viable cells [%]	92 ± 6
	1	necrotic cells [%]	1.2 ± 0.8
		late apoptotic cells [%]	4.9 ± 2.8
		early apoptotic cells [%]	1.3 ± 0.5
		viable cells [%]	93 ± 4
	10	necrotic cells [%]	6.7 ± 3.1
		late apoptotic cells [%]	64 ± 6
		early apoptotic cells [%]	16 ± 1
		viable cells [%]	13 ± 3
**2**	1	necrotic cells [%]	1.4 ± 0.8
		late apoptotic cells [%]	2.0 ± 0.2
		early apoptotic cells [%]	1.1 ± 0.4
		viable cells [%]	95 ± 1
	10	necrotic cells [%]	1.5 ± 0.8
		late apoptotic cells [%]	2.0 ± 0.3
		early apoptotic cells [%]	1.0 ± 0.3
		viable cells [%]	96 ± 1
**3**	1	necrotic cells [%]	1.0 ± 0.5
		late apoptotic cells [%]	2.1 ± 0.3
		early apoptotic cells [%]	1.0 ± 0.7
		viable cells [%]	96 ± 1
	10	necrotic cells [%]	0.72 ± 0.49
		late apoptotic cells [%]	2.1 ± 0.7
		early apoptotic cells [%]	0.7 ± 0.3
		viable cells [%]	97 ± 1

**4**	0.1	necrotic cells [%]	1.7 ± 1.1
		late apoptotic cells [%]	2.1 ± 0.4
		early apoptotic cells [%]	1.1 ± 0.5
		viable cells [%]	95 ± 2
	1	necrotic cells [%]	0.93 ± 0.65
		late apoptotic cells [%]	2.4 ± 1.2
		early apoptotic cells [%]	1.1 ± 0.7
		viable cells [%]	96 ± 2
	10	necrotic cells [%]	3.4 ± 4.0
		late apoptotic cells [%]	8.3 ± 4.9
		early apoptotic cells [%]	1.0 ± 0.4
		viable cells [%]	87 ± 8
**5**	0.1	necrotic cells [%]	1.0 ± 0.5
		late apoptotic cells [%]	2.9 ± 1.5
		early apoptotic cells [%]	3.0 ± 2.2
		viable cells [%]	93 ± 4
	1	necrotic cells [%]	0.85 ± 0.48
		late apoptotic cells [%]	3.3 ± 1.3
		early apoptotic cells [%]	1.0 ± 0.5
		viable cells [%]	95 ± 2
	10	necrotic cells [%]	6.3 ± 2.2
		late apoptotic cells [%]	40 ± 6
		early apoptotic cells [%]	25 ± 7
		viable cells [%]	29 ± 11
**6**	0.1	necrotic cells [%]	17 ± 27
		late apoptotic cells [%]	4.7 ± 2.8
		early apoptotic cells [%]	1.0 ± 0.7
		viable cells [%]	77 ± 29
	1	necrotic cells [%]	1.0 ± 1.3
		late apoptotic cells [%]	2.0 ± 0.7
		early apoptotic cells [%]	1.1 ± 07
		viable cells [%]	96 ± 2
	10	necrotic cells [%]	0.94 ± 0.52
		late apoptotic cells [%]	3.7 ± 0.1
		early apoptotic cells [%]	1.1 ± 0.3
		viable cells [%]	94 ± 0
**L**	10	necrotic cells [%]	0.61 ± 0.44
		late apoptotic cells [%]	1.6 ± 0.2
		early apoptotic cells [%]	1.0 ± 0.5
		viable cells [%]	97 ± 1
	100	necrotic cells [%]	0.86 ± 0.33
		late apoptotic cells [%]	1.9 ± 0.2
		early apoptotic cells [%]	1.0 ± 0.2
		viable cells [%]	96 ± 0

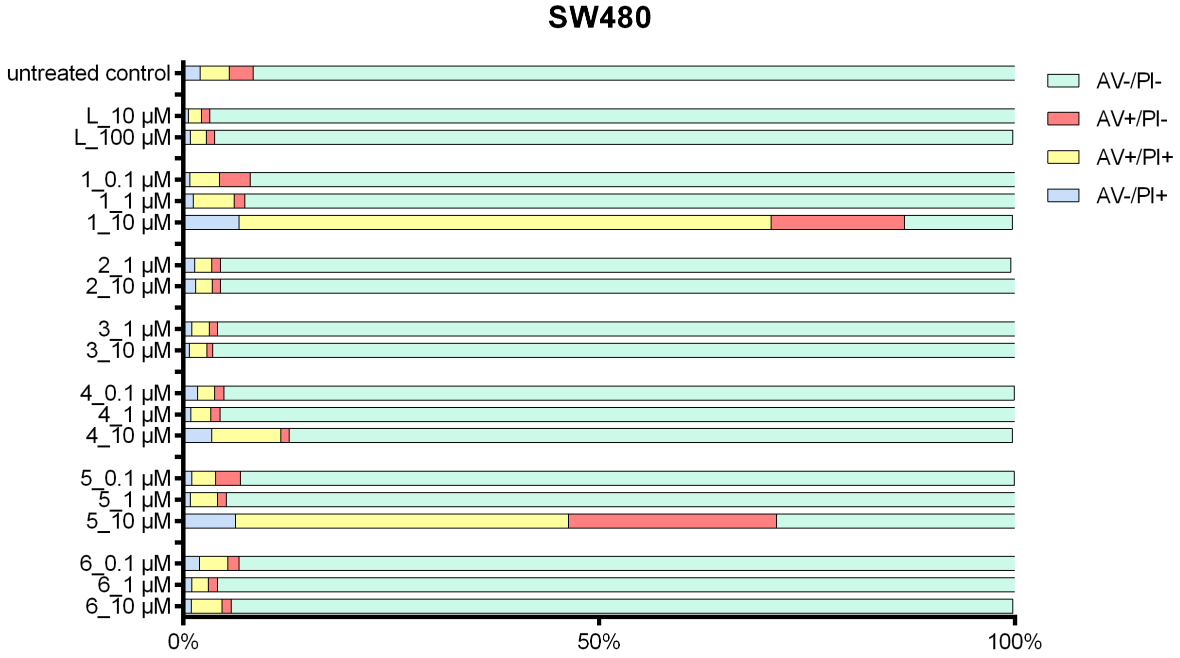

Figure S33: Flow-cytometric analysis of apoptosis/necrosis induction in SW480 cells incubated with different concentrations of **L** and **1–6** for 24 h. Percentages of necrotic (AV-/PI+), late apoptotic (AV+/PI+), early apoptotic (AV+/PI-) and viable (AV-/PI-) cells were determined by double-staining with annexin V-FITC/PI.

ROS assay

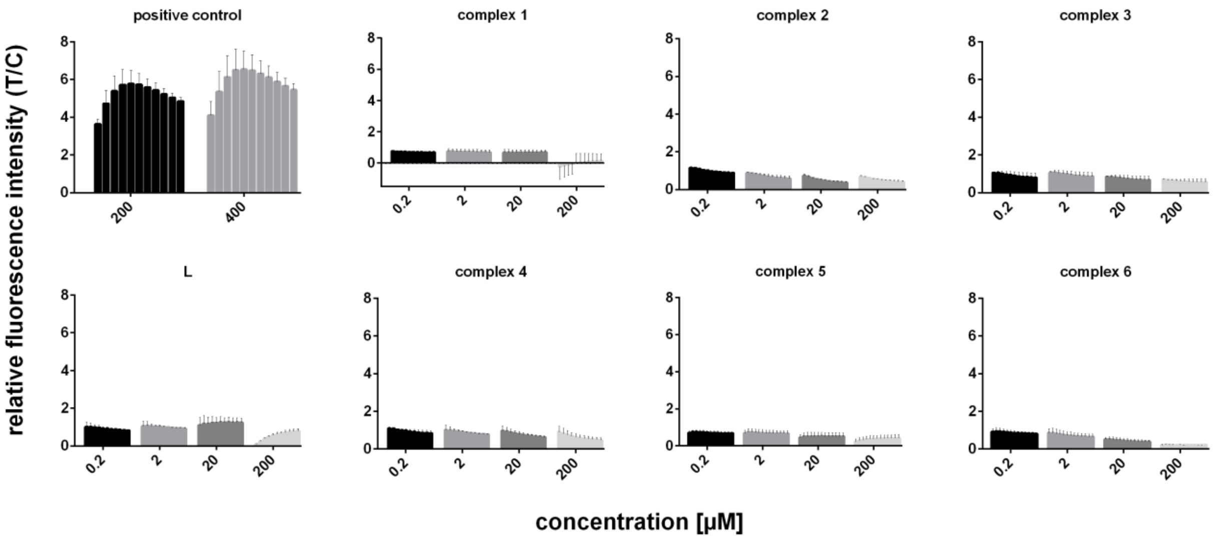

Figure S34: ROS induction in CH1/PA-1 cells by **L** and complexes **1–6** in four different (0.2–200 µM) concentrations and by a positive control (200 and 400 µM tert-butylhydroperoxide) over time for up to 2 h. The values are normalized to the untreated control.

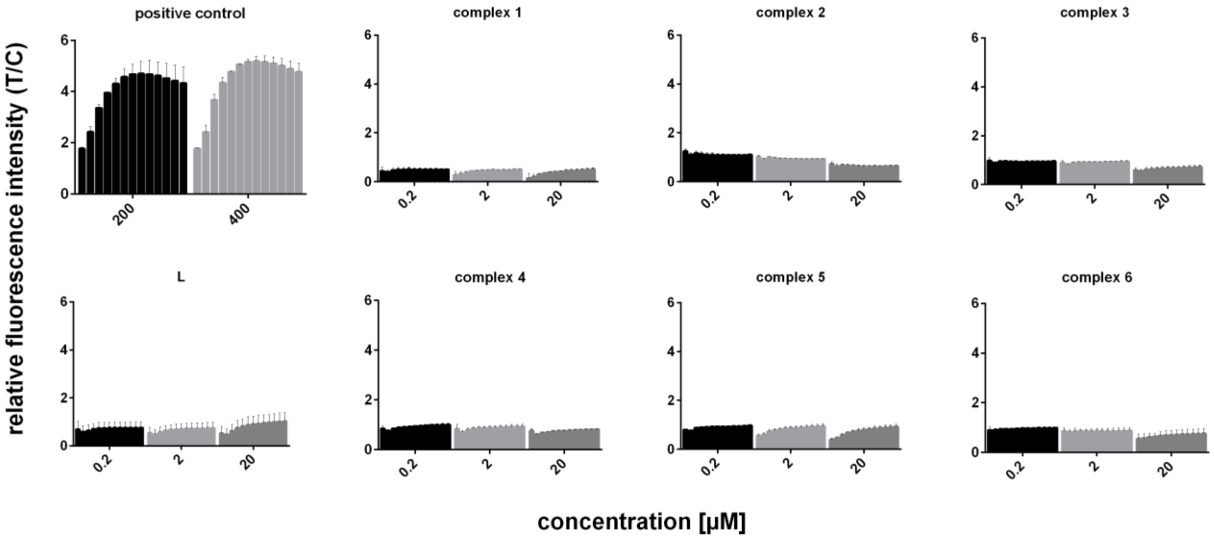

Figure S35: ROS induction in SW480 cells by **L** and complexes **1–6** in four different (0.2–20 µM) concentrations and by a positive control (200 and 400 µM tert-butylhydroperoxide) over time for up to 2 h. The values are normalized to the untreated control.

dsDNA plasmid assay

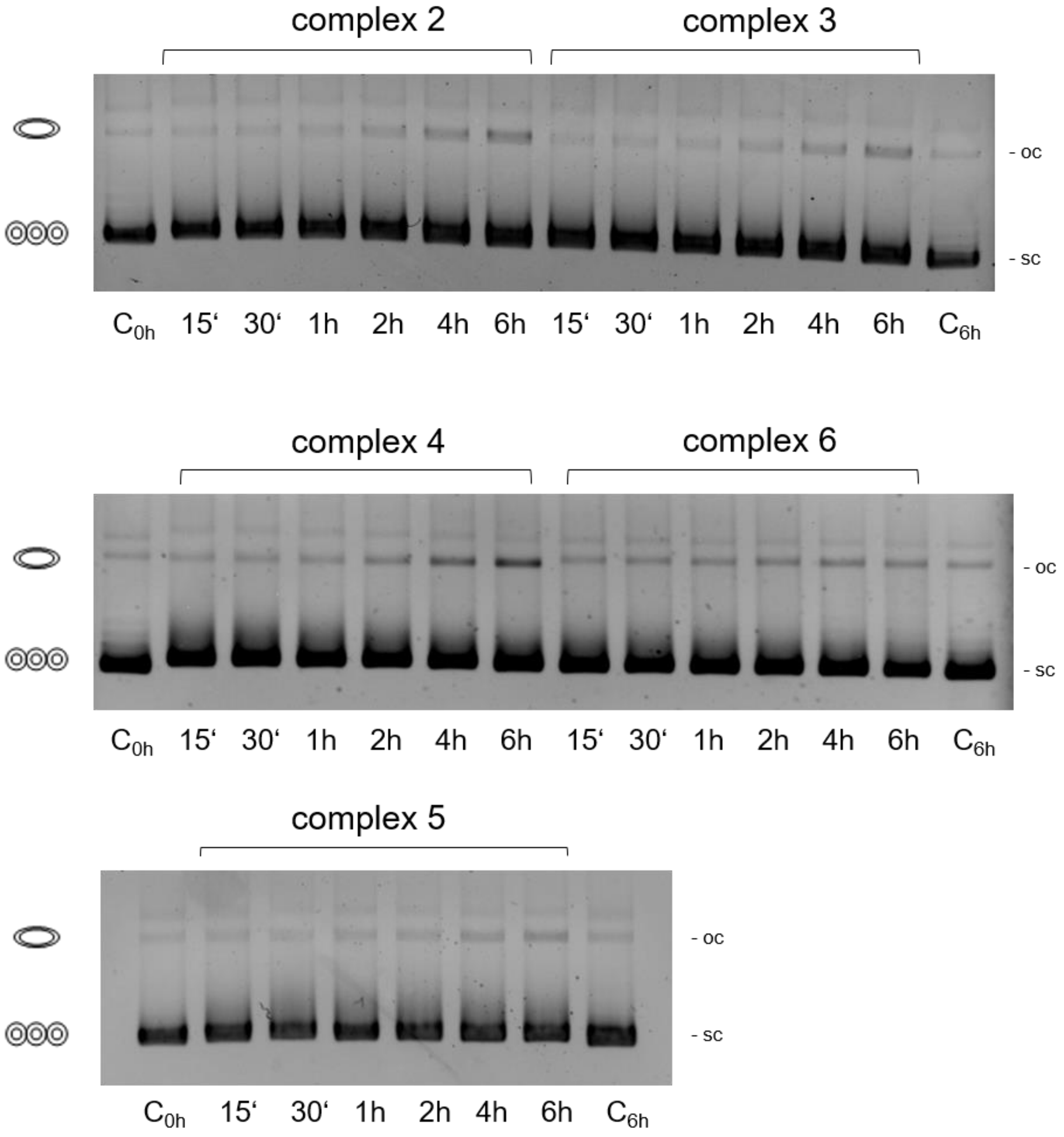

Figure S36: Electropherograms of dsDNA plasmid (pUC19) interactions with 50 μM of compounds **2–6**. The pUC19 plasmid was incubated with the complexes for different time periods (from 15 min to 6 h) at 37 °C and the different plasmid forms were separated by agarose gel electrophoresis. ‘C0h’ corresponds to an untreated, non-preincubated control at the time point of gel loading, while ‘C6h’ corresponds to an untreated, 6 h incubated control; oc, open circular; sc, supercoiled are forms of the plasmid DNA (Electropherograms of **1** and **L** were published previously [8] (as complex **2a** and ligand **2** in Figure S79).

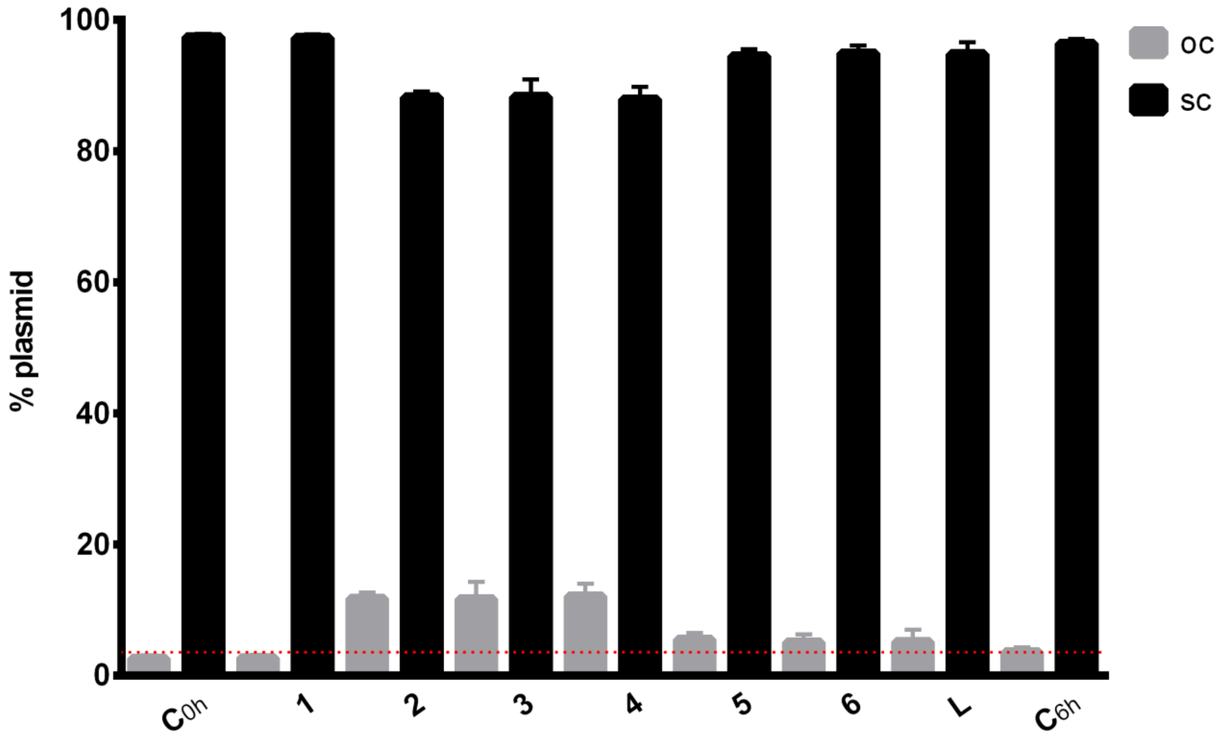

Figure S37: Quantified data from the electrophoretic plasmid DNA interaction studies shown in Figure S36. The grey columns correspond to the open-circular (oc) form and black columns to the supercoiled (sc) form of the pUC19 plasmid after 6 h of incubation and the controls (C0h and C6h). Data for complexes **1** and **L** are taken from [8].

NQO1 activity assay

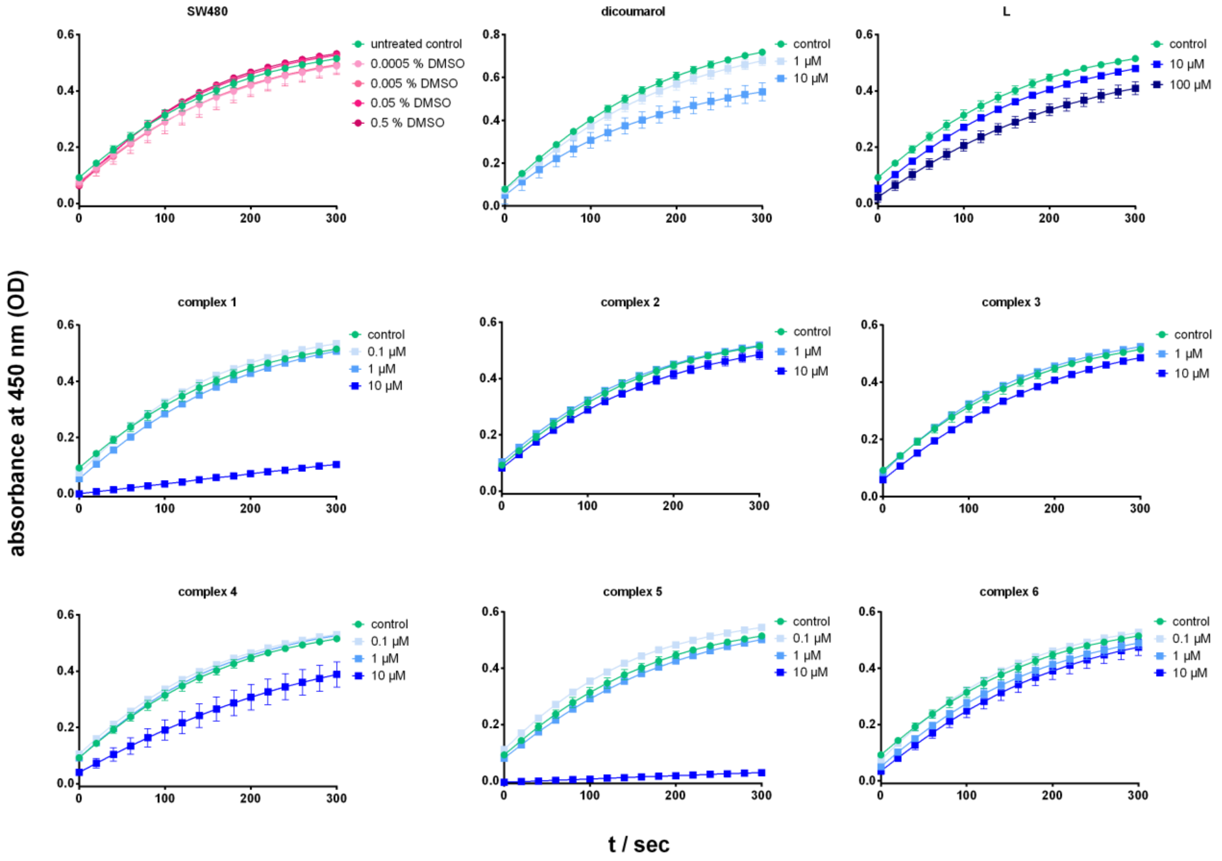

Figure S38: Time-dependent impact on NQO1 activity in SW480 cell extracts after treatment with compounds **L** and **1–6** or dicoumarol (positive control) compared to negative controls with and without DMSO (concentrations corresponding to the content in solutions of the test compounds). Data are expressed as OD (450 nm) per 100 µg/mL of total cellular protein.

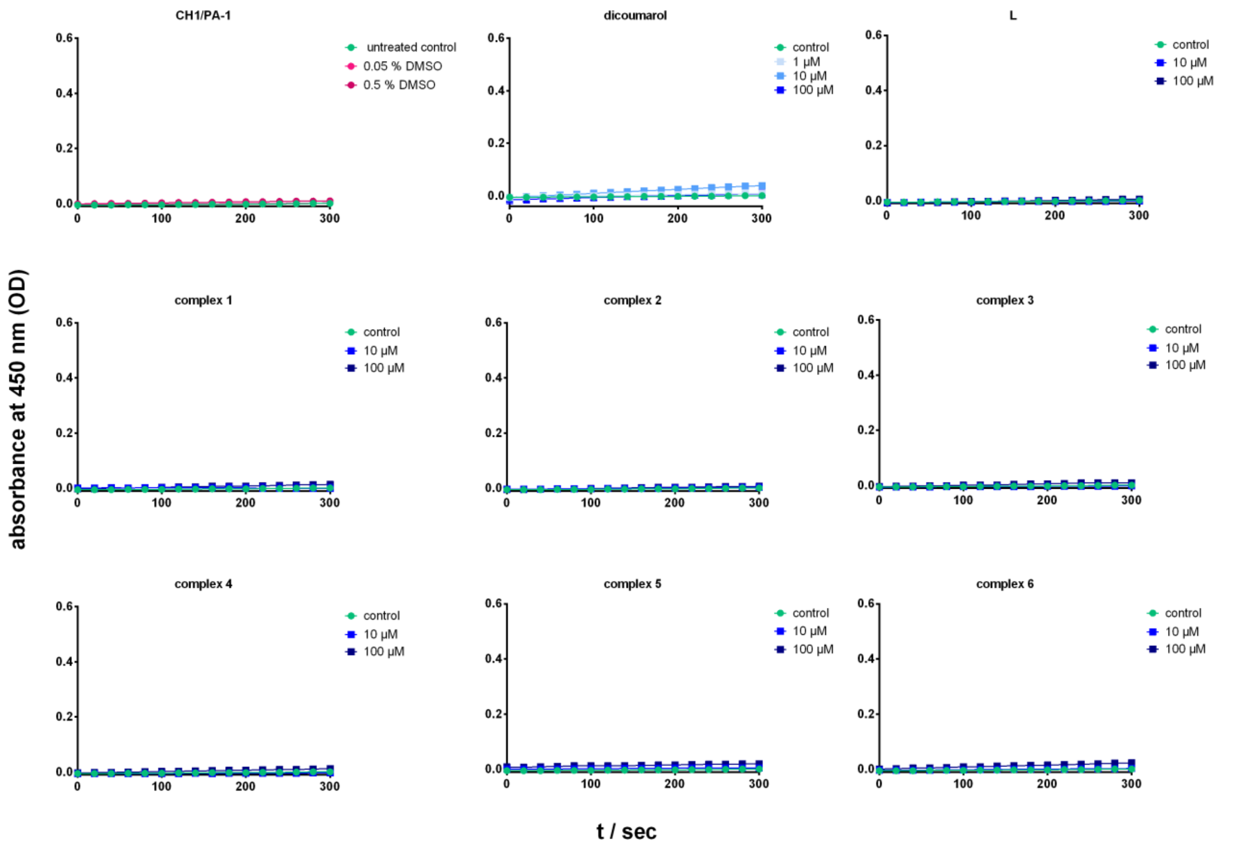

Figure S39: Time-dependent impact on NQO1 activity in CH1/PA-1 cell extracts after treatment with compounds **L** and **1–6** or dicoumarol (positive control) compared to negative controls with and without DMSO (concentrations corresponding to the content in solutions of the test compounds). Data are expressed as OD (450 nm) per 100 µg/mL of total cellular protein.

References

1. AXS, B. *Bruker SAINT* v.838B Copyright(C); Bruker AXS, 2005-2019.
2. Sheldrick, G.M. Sadabs. *University of Göttingen, Germany* **1996**.
3. Dolomanov, O.V.; Bourhis, L.J.; Gildea, R.J.; Howard, J.A.K.; Puschmann, H. OLEX2: a complete structure solution, refinement and analysis program. *Journal of Applied Crystallography* **2009**, *42*, 339-341, doi:doi:10.1107/S0021889808042726.
4. Hubschle, C.B.; Sheldrick, G.M.; Dittrich, B. ShelXle: a Qt graphical user interface for SHELXL. *Journal of Applied Crystallography* **2011**, *44*, 1281-1284, doi:doi:10.1107/S0021889811043202.
5. Sheldrick, G. SHELXS v 2016/4. **2015**.
6. Sheldrick, G. Crystal structure refinement with SHELXL. *Acta Crystallographica Section C* **2015**, *71*, 3-8, doi:doi:10.1107/S2053229614024218.
7. Spek, A. Structure validation in chemical crystallography. *Acta Crystallographica Section D* **2009**, *65*, 148-155, doi:doi:10.1107/S090744490804362X.
8. Geisler, H.; Westermayr, J.; Cseh, K.; Wenisch, D.; Fuchs, V.; Harringer, S.; Plutzar, S.; Gajic, N.; Hejl, M.; Jakupec, M.A.; et al. Tridentate 3-Substituted Naphthoquinone Ruthenium Arene Complexes: Synthesis, Characterization, Aqueous Behavior, and Theoretical and Biological Studies. *Inorg. Chem.* **2021**, *60*, 9805-9819, doi:10.1021/acs.inorgchem.1c01083.
